# Quantum probe hyperpolarisation of molecular nuclear spins

**DOI:** 10.1038/s41467-018-03578-1

**Published:** 2018-03-28

**Authors:** David A. Broadway, Jean-Philippe Tetienne, Alastair Stacey, James D. A. Wood, David A. Simpson, Liam T. Hall, Lloyd C. L. Hollenberg

**Affiliations:** 1 Centre for Quantum Computation and Communication Technology, School of Physics, University of Melbourne, Parkville, VIC 3010 Australia; 2 School of Physics, University of Melbourne, Parkville, VIC 3010 Australia; 3 Melbourne Centre for Nanofabrication, Clayton, VIC 3168 Australia; 4 Department of Physics, University of Basel, Klingelbergstrasse 82, 4056 Basel, Switzerland

## Abstract

Hyperpolarisation of nuclear spins is important in overcoming sensitivity and resolution limitations of magnetic resonance imaging and nuclear magnetic resonance spectroscopy. Current hyperpolarisation techniques require high magnetic fields, low temperatures, or catalysts. Alternatively, the emergence of room temperature spin qubits has opened new pathways to achieve direct nuclear spin hyperpolarisation. Employing a microwave-free cross-relaxation induced polarisation protocol applied to a nitrogen vacancy qubit, we demonstrate quantum probe hyperpolarisation of external molecular nuclear spins to ~50% under ambient conditions, showing a single qubit increasing the polarisation of ~10^6^ nuclear spins by six orders of magnitude over the thermal background. Results are verified against a detailed theoretical treatment, which also describes how the system can be scaled up to a universal quantum hyperpolarisation platform for macroscopic samples. Our results demonstrate the prospects for this approach to nuclear spin hyperpolarisation for molecular imaging and spectroscopy and its potential to extend beyond into other scientific areas.

## Abstract (web summary)

Molecules with ‘hyperpolarised’ nuclear spins can be used to improve MRI performance but require an efficient polarisation method. Broadway et al. demonstrate a quantum control protocol using a nitrogen vacancy centre inside a diamond to hyperpolarise protons within molecules deposited on the surface.

## Introduction

The hyperpolarisation of nuclear spins within target molecules is a critical and complex challenge in magnetic resonance imaging (MRI)^1^ and nuclear magnetic resonance (NMR) spectroscopy^2^. Hyperpolarisation offers enormous gains in signal and spatial resolution which may ultimately lead to the development of molecular MRI and NMR^3^, with the prospect of revolutionising many areas of research and clinical applications. Well-known hyperpolarisation techniques include high-field/low temperature brute-force methods^4^, dynamic nuclear polarisation^6^, optical pumping^5^, and parahydrogen-induced polarisation^7^. On the other hand, the rapid advances in semiconductor spin qubit technology for quantum computing^9,10^ and quantum sensing^11–14^ has opened up the exciting possibility of hyperpolarising nuclear spins at a fundamental level via quantum mechanical protocols^6,8,17–24^. Despite early progress^25,26^, the application of quantum probe technology to this problem still faces a number of significant challenges. To be of practical use, a quantum probe must be capable of polarising a relatively large number of remote nuclear spins external to the probe substrate, ideally under ambient conditions.

In the following, we address these challenges using a quantum spin probe in diamond as an entropy pump, and, using a combined experimental and theoretical analysis, demonstrate polarisation of external molecular spin ensembles to about 50% over relatively large volumes at room temperature, with the prospect of scaling up to a universal hyperpolarisation platform suitable for clinical applications. In contrast to existing methods, our quantum polarisation approach is tunable to a range of nuclear species, is inherently free of radiofrequency (RF) or microwave fields and extraneous chemistry, and is carried out at room temperature.

## Results

### Principle of the technique

**Fig. 1:**
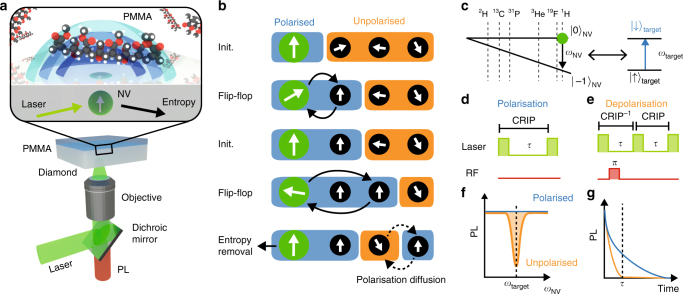
Quantum probe hyperpolarisation of nuclear spin ensembles. **a** Schematic of the system showing a near-surface nitrogen vacancy (NV) spin probe in diamond and a hydrogen nuclear spin target ensemble in molecular Poly(methyl methacrylate) (PMMA) on the surface. The NV probe is initialised by a green laser (532 nm), and read out via its photoluminescence (PL) signal. The shaded blue surfaces denote different regimes of polarisation capabilities arising from the spatial dependence of the nuclear spin coupling to the NV qubit. **b** Schematic of cross relaxation induced polarisation (CRIP) implemented on a spin system illustrating the build up of polarisation from repeated application of the CRIP sequence. Diffusion effects act in competition with the CRIP entropy pumping mechanism, but also allow for polarisation at distances beyond that reachable via the hyperfine interaction. **c** Energy-level diagram of the NV (left) showing the relative positions of various target nuclear spins (right) resonance conditions. **d**, **e** The control sequences (laser pulses in green, RF pulses in red) used for polarising a target spin ensemble using CRIP (**d**) and for controlled depolarisation using the combined CRIP^−1^ × CRIP protocol (**e**). **f** Schematic showing the cross-relaxation spectrum obtained by measuring the PL during the CRIP (blue) or depolarisation (orange) sequence with a constant interaction time *τ*, while scanning the NV frequency *ω*_NV_. **g** Similarly, the cross-relaxation curve is obtained by scanning *τ* with *ω*_N*V*_ set at the resonance

For this study, we employ a nitrogen vacancy (NV) quantum spin probe in a diamond substrate (Fig. 1a)^11^. Existing approaches to NV-based hyperpolarisation involve the use of an external microwave field to tune the NV Rabi frequency to the Larmor frequency of the target spins, thereby enabling rotating-frame cross-relaxation via the *A*_*z*,*x*_ and *A*_*z*,*y*_ components of their hyperfine interaction. Our approach, on the other hand, takes advantage of the greater (*A*_*x*,*y*;*x*,*y*_) NV-target hyperfine interaction associated with direct lab-frame cross-relaxation, which allows for an order of magnitude greater target polarisation rate. This is achieved by using an external magnetic field, *B*, to tune the ground-state spin transition frequency of the NV (*ω*_NV_) into resonance with target nuclear spins (*ω*_n_) (Fig. 1b). For a given target species, the spin resonance condition is fulfilled at a magnetic field B_*_(*γ*_n_) ≈ 2*D*/(*γ*_NV_ − *γ*_n_)^27^, where *γ*_n_, *γ*_NV_ are the target and NV gyromagnetic ratios, and *D* is the NV zero-field splitting (Fig. 1c). Entropy pumping is facilitated by repeated application of the cross relaxation induced polarisation (CRIP) sequence (Fig. 1d), wherein the NV spin is optically initialised into $\left| 0 \right\rangle$ state and the NV-target hyperfine interaction is allowed to occur for a given period of time, *τ* (of order microseconds). The transfer of magnetisation caused by this interaction thus polarises the target spins into their $\left| \downarrow \right\rangle$ state (Fig. 1c). For the sake of comparison, depolarisation may be facilitated by interleaving the initialisation of the NV spin into the opposite state $\left| { - 1} \right\rangle$ by the application of an RF *π*-pulse (Fig. 1e).

To quantitatively understand the effect of the CRIP protocol on the target spin ensemble, we developed a microscopic theory that explicitly includes the dipole interactions of ensemble spins and their interaction with a single NV quantum probe (see Supplementary Note 1 for details). We define the polarisation of a spin at position **R** (relative to the NV) and time *t* to be *P*(**R**, *t*); with the evolution of *P*(**R**, *t*) described by

1 $$\frac{\partial }{{\partial t}}P({\bf{R}},t) = \left( {\beta {\kern 1pt} \nabla ^2 - u({\bf{R}}) - {\mathrm{\Gamma }}_{{\mathrm{SL}}}} \right)P({\bf{R}},t) + u({\bf{R}}),$$

subject to an initial unpolarised state *P*(**R**, *t*) = 0; where *u*(**R**) = *A*^2^(**R**)/2Γ_2_ is the effective cooling coefficient resulting from the hyperfine coupling *A*(**R**) with the NV spin, Γ_2_ is the dephasing rate of the NV spin *β* is the effective polarisation diffusion coefficient related to the intra-target interactions, and Γ_SL_ is the spin-lattice relaxation rate of the target spin ensemble. This formulation allows us to predict and describe the spatial extent of polarisation for a given target sample of arbitrary geometry.

To probe the polarisation effect experimentally, we monitor the spin-dependent photoluminescence (PL) from the NV^28–30^ during the laser pulses, which decays as a function of the CRIP sequence time as $e^{ - \Gamma _{{\mathrm{tot}}}\tau }$. Here Γ_t*ot*_ is the NV longitudinal relaxation rate, which can be expressed as the sum Γ_tot_ = Γ_bg_ + Γ_CR_, where Γ_bg_ is the background rate caused by lattice phonons or surface effects, and Γ_CR_ is due to cross-relaxation. The latter follows a Lorentzian dependence on the detuning between the probe and target transition frequencies^28^,

2 $${\mathrm{\Gamma }}_{{\mathrm{CR}}} = \frac{{A_P^2{\mathrm{\Gamma }}_2}}{{2{\mathrm{\Gamma }}_2^2 + 2\left( {\omega _{{\mathrm{NV}}} - \omega _{\mathrm{n}}} \right)^2}},$$

where $A_P^2$ is the total effective hyperfine field seen by the NV due to the target ensemble, which is related to the polarisation distribution via

3 $$A_P^2 = \frac{{n_{\mathrm{t}}}}{2}{\int} {\left[ {1 - P({\bf{R}},t)} \right]A^2({\bf{R}})\,{\mathrm{d}}^3{\bf{R}}} ,$$

where *n*_t_ is the density of the target spin ensemble. The key indicator of significant polarisation is therefore a reduction in Γ_CR_, which manifests as the disappearance of the target ensemble’s spectral feature from the cross-relaxation spectrum (Fig. 1f), and can be quantified by measuring the cross-relaxation curve at resonance (Fig. 1g).

### Polarisation of internal ^13^C spins

**Fig. 2:**
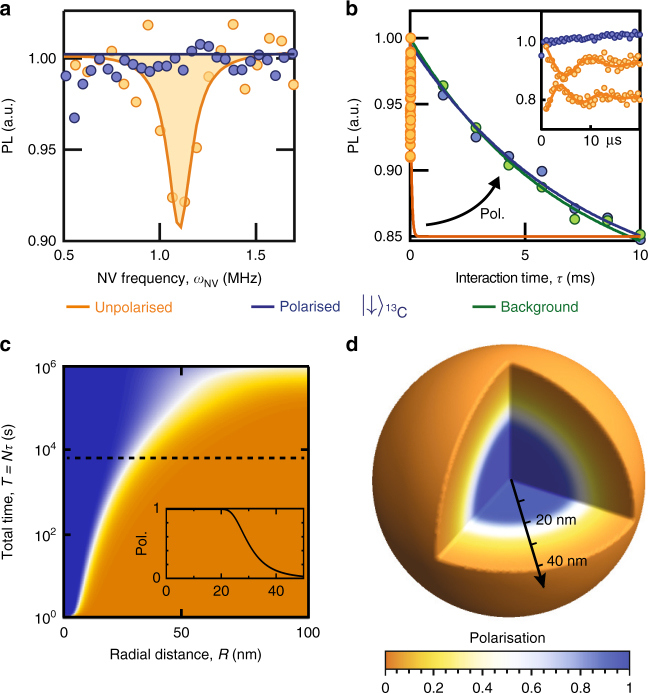
Cross-relaxation induced polarisation of ^13^C spins in diamond. **a** Cross-relaxation spectra of a single NV spin near the ^13^C resonance ($\omega _{{\mathrm{N}}V} = 1.1$ MHz), obtained with an interaction time *τ* = 4 µs using the CRIP sequence (blue) and the depolarisation sequence (orange, only the readout following the NV initialisation in $|0\rangle$ is shown). Sequences were repeated *N* = 10^5^ times at each point. **b** Cross-relaxation curves obtained by increasing *τ* at the ^13^C resonance with the CRIP sequence (blue) and depolarisation sequence (orange), and off-resonance to obtain the background relaxation curve (green). Zoom-in at short times for the polarised (blue) and unpolarised case (orange, top and bottom curves correspond to the NV initialised in $|0\rangle$ and $| - 1\rangle$, respectively). **c** Calculated radial polarisation profiles relative to the NV spin (averaged over all angles), calculated from Eq. (1) for a random 1.1% ^13^C spin ensemble for varying total polarisation times, *T* = *Nτ*. Inset: profile along dashed line, corresponding to *T* = 2 h. **d** Three-dimensional representation of the polarisation distribution at *T* = 2 h

Experimentally, we first demonstrate our technique on the inherent 1.1% ^13^C spin ensemble surrounding a NV probe in the diamond substrate by tuning to the ^13^C resonant condition at B_*_(^13^C) = 1024.9 G. Comparison of the cross-relaxation spectra for CRIP and depolarisation sequences (Fig. 2a) shows the complete removal of the ^13^C resonance peak for interaction times of *τ* = 4 µs, indicating efficient polarisation of the nearest spins, as compared with the target prepared using the depolarising sequence. This is confirmed in the cross-relaxation curves as a function of *τ* (Fig. 2b, inset), where the polarised case shows no evolution of the NV spin state, while the unpolarised case shows coherent flip-flops between the NV and the ^13^C spins.

To investigate the extent of the polarisation effect, we increase the interaction time *τ* so as to be sensitive to more remote ^13^C spins, up to the limit set by the NV centre’s intrinsic spin-phonon relaxation rate, Γ_bg_ ≈ 200 s^−1^. The resulting cross-relaxation curves obtained at the ^13^C resonance using the CRIP and depolarisation sequences are shown in Fig. 2b, from which we extract the total relaxation rate, Γ_tot_. By subtracting Γ_bg_ obtained from the off-resonance relaxation curve (Fig. 2b, green data), we deduce the ^13^C-induced relaxation rate Γ_CR_ = Γ_tot_ − Γ_bg_, which decreases from $\Gamma _{{\mathrm{C}}\mathrm{R}}^{{\mathrm{u}}\mathrm{npol}} \approx 250$ ms^−1^ with the depolarisation sequence, to below the noise floor of the measurement after 5 h of CRIP, $\Gamma _{{\mathrm{CR}}}^{{\mathrm{pol}}}\mathop { < }\limits_ \sim 19$ s^−1^. We use Eq. (1) (with *β* = 0.0335 nm^2^s^−1^ corresponding to the given ^13^C density) to calculate the time dependence of the radial polarisation profile for total polarisation times of 1–10^6^ s, as depicted in Fig. 2c. By relating the spatial polarisation distribution, *P*(**R**,*t*), to the cross-relaxation rate, Γ_CR_, via Eq. (2), we find the theoretical results are consistent with the experiment for polarisation times in excess of two hours (Fig. 2c, dashed line). Examination of the spatial polarisation distribution (Fig. 2c, inset, and Fig. 2d) implies a polarisation level of more than 99% within 21 nm of the NV, equating to a 6 × 10^6^-fold increase on thermal polarisation for 3 × 10^5^ spins.

### Polarisation of external ^1^H spins

With the basic protocol established, we now move to the polarisation of molecular ^1^H nuclear spins external to the diamond crystal. A solution of poly(methyl methacrylate), PMMA, was applied directly to a diamond surface^12^ with single NV spin probes located 8–12 nm below the surface with an average density of 10^8 ^cm^−^^2^. The initial criteria for finding suitable NVs involved finding a trade-off between maximising the NV-H coupling (shallow), and minimising the coupling to sparse electronic surface defects (deep) which limit the NV linewidth and the background *T*_1_ time. The surface is also known to retain a 1–2 nm thick layer of protons of density commensurate with that of PMMA^15^, which we therefore regard as being part of the target proton system to be polarised.

**Fig. 3:**
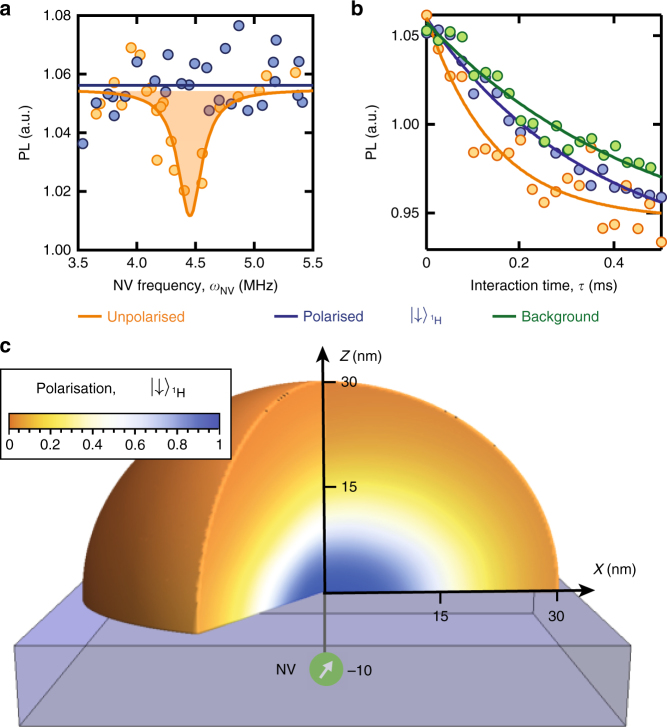
Polarisation of external molecular ^1^H spins. **a** Cross-relaxation spectra near the ^1^H resonance (*ω*_NV_ = 4.4 MHz), for a single NV spin located 10 nm below a PMMA layer, obtained using *τ* = 20 μs and *N* = 10^5^ with the CRIP sequence (blue) and the depolarisation sequence (orange). **b** Cross-relaxation curves obtained by increasing *τ* at the ^1^H resonance with the CRIP sequence (blue) and depolarisation sequence (orange), and off-resonance to obtain the background relaxation rate (green). **c** Three-dimensional representation of the ^1^H spin polarisation distribution in PMMA on the surface of a diamond (blue block), calculated from Eq. (1) in the steady state

CRIP was applied with the external magnetic field tuned to resonance at B_*_(^1^H) = 1026.2 G. With a much higher diffusion constant and spin-lattice relaxation rate (*β* = 781 nm^2^ s^−1^, Γ_SL_ = 1s^−1^)^16^ relative to the intrinsic ^13^C case, the ^1^H system effectively reaches steady-state within a few seconds. Application of the CRIP sequence to NV1 (data for other NVs are shown in the Supplementary Note 2) for *τ* = 20s (Fig. 3a) shows the removal of the hydrogen spectral feature (blue), as compared with the depolarising sequence (orange). From the cross-relaxation curves after 1 h of CRIP (Fig. 3b), we extract ^1^H-induced rates for the unpolarised ($\Gamma _{{\mathrm{C}}\mathrm{R}}^{{\mathrm{u}}\mathrm{npol}}$) and polarised ($\Gamma _{{\mathrm{C}}\mathrm{R}}^{{\mathrm{p}}\mathrm{ol}}$) PMMA ^1^H spin ensembles to be 2.71 ms^−1^ and 0.96 ms^−1^, respectively. The ratio $\Gamma _{{\mathrm{CR}}}^{{\mathrm{unpol}}}/\Gamma _{{\mathrm{CR}}}^{{\mathrm{pol}}} = 2.8(3)$ (consistent with the value of 2.4(3) obtained using another NV), is in good agreement with the solution to Eq. (1) for the PMMA ensemble, which gives a ratio of 2.2 in the steady state. The corresponding spatial polarisation distribution is shown in Fig. 3c, indicating that the system reaches 50% average polarisation over a volume of ~ (26 nm)^3^. Thus, we conclude that the single spin quantum probe has increased the average polarisation of roughly a million hydrogen spins by some six orders of magnitude over the room temperature Boltzmann thermal background.

### Improvements and scaling up

**Fig. 4:**
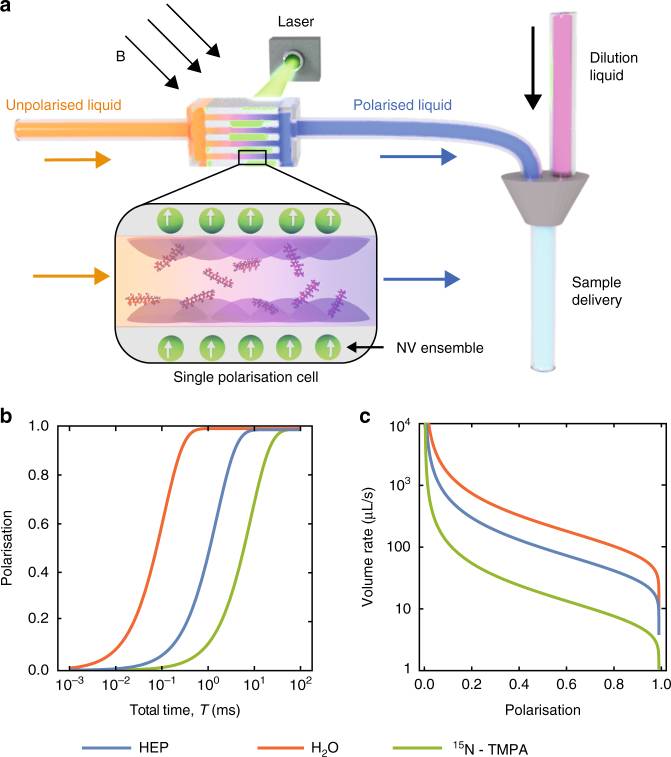
Universal MRI contrast agent hyperpolarisation platform. **a** Schematic of a quantum polarisation stack comprising multiple diamond membranes, each containing NV array layers on both sides, in a homogeneous magnetic field tuned to the nuclear gyromagnetic ratio of the target agent spin species. The unpolarised agent in concentrated solution (orange) flows into the stack channels, where the liquid is polarised through the application of CRIP (via a pulsed laser). The out-flowing polarised liquid (blue) is then diluted for use. Zoomed schematic shows a single polarisation cell comprising a channel formed by dual diamond membranes each with a near-surface NV layer. **b** Average polarisation level from a single polarisation cell, for various targets (HEP, H_2_O, and ^15^N-TMPA), calculated for varying polarisation times assuming perfect mixing of a 1 M target agent solution with a cell height of 1 μm. **c** Outflow rate (after dilution to 1 mM for application delivery) from 10 polarisation cells at different levels of polarisation

There is scope for improvement on these proof-of-concept results: for example, engineering NV depths to 5 nm would increase the rate of target spin polarisation by an order of magnitude, and improvements in the inherent NV dephasing rate Γ_2_ (e.g. via improved surface properties) will allow for more precise tuning to different nuclear spin species. As the protocol is all optical, scaling up for high-volume production could be achieved by stacking multiple NV arrays (Fig. 4a) and/or increasing the effective interaction area via surface patterning^31^, however, issues such as the creation and control of homogeneous DC magnetic fields over the spatial extent of the ensemble are ongoing endeavours that are beyond the scope of the work presented here.

The results presented here indicate that the CRIP protocol could produce macroscopic quantities of MRI contrast agents with high polarisation levels. For example, we consider ^13^C isotopically enriched HEP (hydroxyethylpropionate,^13^C_5_H_10_O_3_), a well-known MRI contrast agent^32^. Using a single hyperpolarisation cell comprised of two NV arrays in diamond membranes separated by 1 μm (see zoomed schematic in Fig. 4a; we assume an NV density of 4 × 10^11^ cm^−2^ over a 4 × 4 mm diamond surface^33^), the rate of polarisation transfer to a concentrated 1 M precursor HEP solution is 4 μL s^−1^ at a polarisation level of 80%. The polarisation levels for different contrast agents in 1 M precursor solutions are plotted against polarisation time (assuming perfect mixing occurs over these timescales) in Fig. 4b. In Fig. 4c, we plot the final delivery rate after dilution to 1 mM for a stack of 10 cells, showing that delivery rates of order 100 μL s^−1^ for clinical applications^34^ are achievable.

## Discussion

In summary, we have experimentally demonstrated hyperpolarisation of molecular nuclear spins under ambient conditions by employing a quantum spin probe entropy pump. The technique works at low magnetic fields and room temperature conditions, requires no RF or microwave fields, and operates directly on the target molecules without the need for catalysts or free radicals. With high polarisation rates and tunability, there are excellent prospects for scale-up of the system to produce macroscopic quantities of a range of contrast agents at polarisation levels required for molecular MRI/NMR. The technique can be extended to other nuclear spin species and may also provide pathways in quantum information for initialisation of quantum simulators, or increasing the fidelity of operations through spin-bath neutralisation. Future work will involve external verification of nuclear spin polarisation using conventional NMR techniques.

## Methods

### Experimental apparatus

The experimental apparatus consists of a custom-built confocal microscope and a permanent magnet mounted on a scanning stage, the same setup used, and described, in^29^. In summary, the excitation source is a solid-state laser emitting at a wavelength *λ* = 532 nm (Laser Quantum Gem 532). The objective lens (Olympus UPlanSApo 100×, NA = 1.4 Oil) is mounted on an XYZ scanning stage (PI P-611.3 NanoCube) to allow fast laser scanning. The PL emitted by the diamond sample is separated from the laser light using a dichroic beam splitter and a band-pass filter, and coupled into a multimode fibre connected to a single photon counting module (Excelitas SPCM-AQRH-14-FC). For *T*_1_ measurements, the laser beam is modulated by an acousto-optic modulator (AA Opto-Electronic MQ180-A0,25-VIS) in a double pass configuration, and the PL signal is analysed by a time digitiser (FastComTec P7889). For optically detected magnetic resonance (ODMR) measurements, a 20-μm copper wire is spanned on the surface of the diamond and connected to the output of a microwave generator (Agilent N5181A) modulated by a switch (Mini-Circuits ZASWA-2-50DR+). Laser and microwave modulations are controlled by a programmable pulse generator (SpinCore PulseBlasterESR-PRO 500 MHz). The magnetic field direction and strength were varied by using a permanent magnet affixed to a set of three linear translation stages (PI M-511) allowing XYZ position control. These stages had a resolution of 100 nm which is sufficient to tune the NV into resonance and align along the field along the NV axis, thus avoiding any misalignment issues^27^.

### Diamond samples

The sample (#132) used for the ^13^C measurements was a $\left\langle {111} \right\rangle$-oriented single crystal, electronic grade, chemical vapour deposition (CVD), 100 μm thick diamond purchased from Delaware Diamond Knives. For the measurements reported in Fig. 2, we used native (as grown) NV centres located far (several μm) from the surface. The sample (#122) used for the ^1^H measurements is a $\left\langle {100} \right\rangle$-oriented single crystal CVD overgrown on a HPHT substrate from Element Six. The overgrowth is roughly 50 μm and electronic grade. The sample has been implanted with ^15^N and ^14^N at 3 keV at a density of 5 × 10^8^ cm^−2^ each. The sample was annealed at 950 °C for 2 h and was exposed to a soft O_2_ plasma for 1 minute^36^. The PMMA was baked onto the surface of the diamond by a heat gun at 85° for 40 min. The diamond has a range of near-surface NV depths (~3–13 nm) determined by NV-NMR spectroscopy using the dynamical decoupling method^35^.

### General acquisition procedure

The spectra shown in Figs. 2 and 3 were obtained as follows. The magnetic field was first aligned along the NV axis by maximising the PL intensity^30,37,38^. When doing this near the GSLAC, we estimate an alignment to less than 0.1°. The magnet was then stepped along the NV direction to vary the magnetic field strength *B*_0_. For each magnet position, an ODMR spectrum was recorded for about 1 minute from which we extract *ω*_NV_ via a Lorentzian fit, before the CRIP sequence is applied. The latter consists of a series of 3-μs laser pulses, sufficient to completely initialise the NV spin state, separated by a wait time *τ*. The signal plotted in Figs. 2a and 3a corresponds to the PL intensity integrated over the first 300 ns of the laser pulse, normalised by the intensity integrated over the last 300 ns. To compare with the non-polarised case, we repeated the scan but by interleaving CRIP and CRIP^−1^ pulse sequences, which acts to prevent polarisation build up in either direction. The CRIP^−1^ simply adds a radiofrequency (RF) pulse to flip the NV spin from |0〉 to |−1〉. This *π* pulse was applied 1 μs after the end of the laser pulse, was 300 ns in duration, and was followed by a wait time *τ* identical to that used in the preceding CRIP sequence. With the interleaved sequence, there are two independent PL readout, one during the laser pulse following the CRIP sequence, the other following the CRIP^−1^ sequence. Note that the interleaved sequence continuously polarises the bath but each pulse flips the direction of this polarisation. As such, because of pulse imperfections it may still partly polarise nearby spins.

For the full-length *T*_1_ measurements presented in main text Figs. 2b and 3c, a similar procedure was applied, except that the magnet was not moved throughout the acquisition, and instead the wait time *τ* was continuously swept. To monitor magnetic field drifts caused by thermal fluctuations, we recorded an ODMR spectrum at regular intervals. We can thus ensure that the NV remains on resonance with the target transition, within the NV linewidth (see details in Supplementary Note 2).

### Model Hamiltonian

Given the generality of our approach with respect to the target species of nuclear spin, we refer to a general environmental target spin system, E. Assuming alignment of the external field with the NV axis, the Hamiltonian becomes

4 $$\begin{array}{l}{\cal H} = 2\pi D{\cal S}_z^2 + \gamma _{{\mathrm{NV}}}B_0S_z + \mathop {\sum}\limits_j \left[ {S \cdot A_j \cdot I_j - \gamma _{\mathrm{n}}B_0I_z^{(j)}} \right] + \mathop {\sum}\limits_{k > j} I_j \cdot B_{jk} \cdot I_k,\end{array}$$

where *S*_*x*,*y*,*z*_ are the Pauli spin matrices of the spin-1 system of the NV, *D* = 2.87 GHz is the corresponding zero-field splitting, *B*_0_ is the external field strength, *γ*_NV_ and *γ*_n_ are the gyromagnetic ratios of the NV and target spins, $I_{x,y,z}^{(j)}$ are the Pauli spin matrices of nuclear spin *j*, *A*_*j*_ is the hyperfine tensor describing the spin-spin interaction between the NV and spin *j*, *B*_*jk*_ is the tensor describing the magnetic dipole interaction between spins *j* and *k*; and summation over *j*,*k* refer to all spins in the environment. In Eq. 4, *γ*_NV_ is defined positive, while *γ*_n_ can be positive or negative depending on the species considered.

### Continuum description

Solving Eq. 4 for *N*_S_ spins, each having hyperfine coupling *A*_*j*_ to the NV, mutual dipole couplings *B*_*jk*_, and initial state of

5 $$\rho (0) = (\left| 0 \right\rangle \left\langle 0 \right|)_{{\mathrm{NV}}}\mathop { \otimes }\limits_j (p_j\left| \uparrow \right\rangle \left\langle \uparrow \right| + (1 - p_j)\left| \downarrow \right\rangle \left\langle \downarrow \right|)_{{\mathrm{t}}_{\mathrm{j}}},$$

the population of the NV $\left| 0 \right\rangle$ state is at a minimum when *t* = *τ* = 2^1/2^*π*/*A*_0_, and is given by

6 $$p_{{\mathrm{NV}}}(\tau ) = 1 - \mathop {\sum}\limits_j \frac{{A_j^2}}{{A_0^2}}p_j(0),$$

where $A_0^2 \equiv \mathop {\sum}\nolimits_j A_j^2$ is the total hyperfine coupling field, and $A_j = \sqrt{ \left( A_{xx}^{(j)}-A_{yy}^{(j)}\right)^2 + \left(2A_{xy}^{(j)}\right)^2 }$.

The change in probability of spin *j* is given by

7 $$\Delta p_j = - p_j\frac{{A_j^2}}{{A_0^2}} - \mathop {\sum}\limits_k \left( {p_j - p_k} \right)\left( {\frac{\tau }{2}\left| {B_{12}} \right| + \frac{{C_{jk}^2}}{{A_0^2}}} \right),$$

where *B*_*jk*_ is the secular magnetic dipole coupling between spins *j* and *k*, and $C_{jk}^2 \equiv A_j^2A_k^2/A_0^2$ is the effective hyperfine-mediated diffusion strength.

In modelling these systems, we typically consider regions of at least 200 nm in size, which means of order $N_{\mathrm{S}} \sim 10^{10}$ spins and ~10^20^ couplings for the case of PMMA, thereby making modelling of discrete spin states computationally unfeasible. We therefore map Eq. 7 to a temporally and spatially continuous

8 $$p_j \mapsto p\left( {{\bf{R}},t} \right).$$

In the discrete description, the probability of finding spin *j* in its $\left| \uparrow \right\rangle$ state is monitored at discrete multiples of the optimal dark time, *τ*. As we are interested in times $t \gg \tau$, we map changes in *p*_*j*_ over time to

9 $$\Delta p_j \mapsto \tau \frac{\partial }{{\partial t}}p\left( {{\bf{R}},t} \right)$$

To obtain the continuum description of the spatial evolution, we put $p_j \mapsto p\left( {{\bf{R}},t} \right)$ and $p_k \mapsto p\left( {{\bf{R}} + {\bf{r}},t} \right)$, and discretise the Laplacian operator to get an effective diffusion term with constant *β*

10 $$\mathop {\sum}\limits_k \left( {p_j - p_k} \right)B_{jk} \mapsto \beta \nabla ^2p\left( {{\bf{R}},t} \right).$$

We also map the discrete hyperfine couplings to a continuous field whose strength is determined by its position relative to the NV, **R**.

11 $$A_j \mapsto A\left( {\bf{R}} \right).$$

The summation over all hyperfine couplings is then mapped to an integral,

12 $$A_0^2 \mapsto n_{\mathrm{t}}{\int} A^2\left( {\bf{R}} \right)\,{\mathrm{d}}^3{\bf{R}}.$$

Finally, to account for additional sources of environmental spin-lattice relaxation, where applicable, we add an additional probability sink in which any probability outside equilibrium decays at a rate Γ_SL_ = 1/*T*_SL_, given by

13 $$R_{{\mathrm{SL}}} = - \Gamma _{{\mathrm{SL}}}\left( {p\left( {{\bf{R}},t} \right) - \frac{1}{2}} \right).$$

The differential equation describing the evolution of this system is thus given by

14 $$\frac{\partial}{\partial t} p({\bf{R}},t) = - u({\bf{R}})p\,({\bf{R}},t) + \beta \nabla ^2p({\bf{R}},t)- \Gamma _{{\mathrm{SL}}}\left( {p\left( {{\bf{R}},t} \right) - \frac{1}{2}} \right),$$

where

15 $$u({\bf{R}}) = \frac{{A^2({\bf{R}})}}{{\tau A_0^2}}$$

is the effective source, or cooling, coefficient at position **R** relative to the NV, Recasting Eq. 14 in terms of the polarisation *P*(**R**, *t*) = 1 − 2*p*(**R**, *t*), we obtain Equation (1) of the main text.

For cases in which the dephasing rate of the NV spin, Γ_2_, is greater than the hyperfine coupling to the desired target, i.e. $\Gamma _2 \gg A_0$, the population of $\left| 0 \right\rangle$ state of the NV spin will exhibit an exponential time dependence. As such, for this regime we instead make the replacement

16 $$u\left( {\bf{R}} \right) = \frac{{A^2({\bf{R}})}}{{2{\mathrm{\Gamma }}_2}},$$

where the details of this process are discussed in the Supplementary Note 1.

Experimentally, Γ_2_ is obtained via the *T*_1_-relaxometry spectrum (with the interleaved sequence), since Γ_2_ defines the width of the cross-relaxation resonances^28^. We note that Γ_2_ is expected to decrease upon polarisation of the ^13^C bath. However, the effect is relatively small (see section Supplementary Note 1), and as such in the calculations shown in main text Fig. 2 Γ_2_ is kept constant and equal to the off-resonance value (i.e. unpolarised case). For the ^1^H case, the dephasing is dominated by surface effects, and is therefore unaffected by the ^1^H polarisation.

Upon solution of Eq. 14 for the polarisation *P*(**R**, *t*), we may determine the total number of spins polarised at time *t*:

17 $$N_{{\mathrm{pol}}}(t) \equiv n_{\mathrm{t}}{\int} P\left( {{\bf{R}},t} \right){\kern 1pt} {\mathrm{d}}^3{\bf{R}}.$$

### Data availability

The data that support the findings of this study are available from the corresponding author upon request.

## Electronic supplementary material

Supplementary Information
