# Supplementary material for: Quantum probe hyperpolarisation of molecular nuclear spins
Source: Nat Commun. 2018 Mar 28;9:1246. doi: 10.1038/s41467-018-03578-1 (PMC5871805; doi:10.1038/s41467-018-03578-1)
Supplement: Supplementary file 1 — Supplementary Information [file 41467_2018_3578_MOESM1_ESM.pdf]

Supplementary Note 1. THEORETICAL BACKGROUND

Hamiltonian of the system

The Hamiltonian describing the coupling of an NV-^{13}C-^{1}H system is given by

$$\mathcal{H} = \mathcal{H}_{\rm NV} + \mathcal{H}_{\rm C13} + \mathcal{H}_{\rm H} + \mathcal{H}_{\rm NV-C13} + \mathcal{H}_{\rm NV-H} + \mathcal{H}_{\rm C13-H}, \quad (1)$$

where $\mathcal{H}_{\rm NV}$, $\mathcal{H}_{\rm C13}$, and $\mathcal{H}_{\rm H}$ describe the self-Hamiltonians of the NV, ^{13}C, and external ^{1}H spin systems, respectively; and the remaining three terms describe the interactions between these systems. In general, the system described by Eq. 1 has no closed form solution. In what follows however, we develop a theoretical framework that accurately describes the polarisation process for the special cases presented in the main text.

To simplify this system, we typically ignore the $\mathcal{H}_{\rm C13-H}$ term on the basis that the nuclear-nuclear interactions between the two systems are intrinsically weak, and made more so by the fact that are significantly detuned from mutual resonance by about 3.3 MHz at our operating points around 1024 G. Further simplifications are made according to which system is being targeted: polarisation of the ^{13}C bath was carried out for deep NVs ($h \sim 10\,\mu$m) with no surface PMMA, hence $\mathcal{H}_{\rm NV-H} \sim 0$; alternatively if a near-surface NV spin is tuned to resonance with the ^{1}H system, it will be detuned from ^{13}C by 3.3 MHz, which is significantly larger than their measured ~ 500 kHz linewidth, hence $\mathcal{H}_{\rm NV-C13} \sim 0$. Thus the theoretical approach to modelling these two systems in response to CRIP is the same for both cases, and will be developed below independent of the choice of the intended system to be polarised.

The overarching principle of this work is to bring the chosen environment into resonance with the NV centre via precise control of an external magnetic field aligned with the NV axis. As the NV may be optically initialised in its $|0\rangle$ state, any proximate unpolarised environmental spins (of which there will initially be many) will absorb the polarisation of the NV spin via their mutual hyperfine interaction. This polarisation will then diffuse to distant environmental spins via their magnetic dipole interactions. By repeating this process many times over, we may produce polarised regions surrounding the NV of up to a few tens of nanometres in size.

Given the generality of our approach with respect to the target species of nuclear spin, we refer to a general environmental target spin system, E. Assuming alignment of the external field with the NV axis, the Hamiltonian becomes

$$\begin{aligned} \mathcal{H} = & \, 2\pi D \mathcal{S}_z^2 + \gamma_{\rm NV} B_0 \vec{S}_z \\ & + \sum_j \left[\vec{S} \cdot \vec{A}_j \cdot \vec{I}_j - \gamma_{\rm n} B_0 \vec{I}_z^{(j)} \right] \\ & + \sum_{k>j} \vec{I}_j \cdot \vec{B}_{jk} \cdot \vec{I}_k, \end{aligned} \quad (2)$$

where $\vec{S}_{x,y,z}$ are the Pauli spin matrices of the spin-1 system of the NV, $D = 2.87$ GHz is the corresponding zero-field splitting, B_0 is the external field strength, $\gamma_{\rm NV}$ and $\gamma_{\rm n}$ are the gyromagnetic ratios of the NV and target spins, $\vec{I}_{x,y,z}^{(j)}$ are the Pauli spin matrices of nuclear spin j, $\mathbf{A}_j$ is the hyperfine tensor describing the spin-spin interaction between the NV and spin-j, $\mathbf{B}_{jk}$ is the tensor describing the magnetic dipole interaction between spins j and k; and summation over j, k refer to all spins in the environment. In Eq. 2, $\gamma_{\rm NV}$ is defined positive, while $\gamma_{\rm n}$ can be positive or negative depending on the species considered.

Cross-relaxation resonances

Transforming to the interaction picture and applying the rotating wave approximation to the NV-target resonances, we may restrict ourselves to the NV subspace spanned by $\{|0\rangle, |-1\rangle\}$, since the $|0\rangle \leftrightarrow |+1\rangle$ transition is detuned from the nuclear spin transitions by approximately 5.7 GHz. We thus define the $|0\rangle \leftrightarrow |-1\rangle$ NV transition frequency as $\omega_{\rm NV} \equiv 2\pi D - \gamma_{\rm NV} B_0$, and the target transition frequency as $\omega_{\rm n} \equiv \gamma_{\rm n} B_0$. The cross-relaxation resonances occur when $|\omega_{\rm NV}| = |\omega_{\rm n}|$. Since $\omega_{\rm NV}$ changes sign at the NV ground state level crossing at $B_0 = 2\pi D/\gamma_{\rm NV} \approx 1024$ G, there are two resonance points before and after the crossing, at magnetic fields $B_<(\gamma_{\rm n}) \sim 2D/(\gamma_{\rm NV} + \gamma_{\rm n})$ and $B_>(\gamma_{\rm n}) \sim 2D/(\gamma_{\rm NV} - \gamma_{\rm n})$, respectively. Here and throughout the paper, we assume $\gamma_{\rm n} > 0$. A negative sign would simply swap the two resonances and their associated NV-target interaction strengths, relative to the crossing.

Near the NV level crossing, hyperfine interaction of the NV electron spin with its own intrinsic nuclear spin (associated with the nitrogen atom) causes the NV levels to mix and anti-cross, adding small corrections to the above

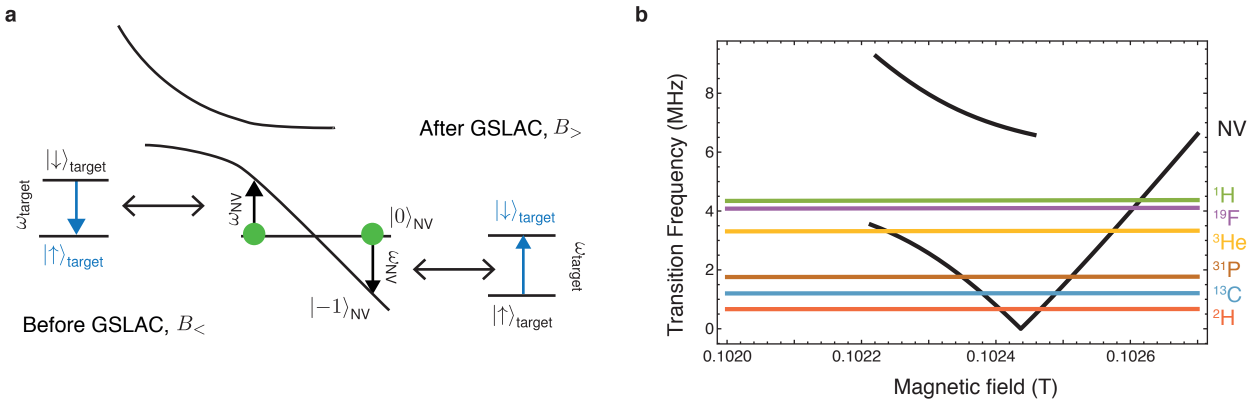

Supplementary Figure 1. **Resonance position of NV and target nuclear spins.** **a** Simplified depiction of the energy levels of the NV spin at the GSLAC as a function of the applied magnetic field, and the polarisation transfer mechanism when on resonance with a target nuclear spin. **b** Transition frequencies as a function of the applied magnetic field, showing the NV-nuclear spin resonance conditions for multiple different nuclear spin species. The black lines represent the two allowed NV transitions which act as the probe frequency.

resonance conditions. A detailed description of this NV ground state level anti-crossing (GSLAC) may be found in Supplementary Ref. [1]. The relevant energy levels of the NV electron spin and target nuclear spin are depicted in Supplementary Fig. 1**a**. As the polarisation transfer is mediated by the NV-target hyperfine interaction, the resulting target spin state ($|\uparrow\rangle$ or $|\downarrow\rangle$) depends on which nuclear spin transition the NV spin is tuned to. Before the GSLAC (e.g. at $B_<(^{13}\text{C}) = 1023.9$ G), CRIP will polarise nuclear spins into the $|\uparrow\rangle$ state (Supplementary Fig. 1**a**, left side). On the other hand, when applied at the resonance condition after the GSLAC (e.g. $B_>(^{13}\text{C}) = 1024.9$ G, or $B_>(^1\text{H}) = 1026.2$ G) the target is polarised into the $|\downarrow\rangle$ state (Supplementary Fig. 1**a**, right side). The NV-nuclear resonance conditions for various species of nuclear spins is shown are Supplementary Fig. 1**b**. One can see that some target nuclear spin species, including ^{1}H for example, do not have a resonance condition before the GSLAC, as the requisite NV spin transition is forbidden. After the GSLAC, however, there is no significant state mixing, which allows all spin species to be addressed via the CRIP protocol. In the following, we will consider only cases where state mixing is negligible, so that we do not have to include the NV nuclear spin in the model.

Definitions

Under this simplification the hyperfine component of the Hamiltonian becomes

$$\vec{\mathcal{S}} \cdot \mathbf{A}_j \cdot \vec{\mathcal{I}} = A^{(j)}_{xx}\mathcal{S}_x\mathcal{I}^{(j)}_x + A^{(j)}_{xy}\left(\mathcal{S}_x\mathcal{I}^{(j)}_y + \mathcal{S}_y\mathcal{I}^{(j)}_x\right) + A^{(j)}_{yy}\mathcal{S}_y\mathcal{I}^{(j)}_y + A^{(j)}_{zz}\mathcal{S}_z\mathcal{I}^{(j)}_z, \tag{3}$$

which may be written more compactly for near-resonant cases before and after the GSLAC as

$$\begin{aligned}
\vec{\mathcal{S}} \cdot \mathbf{A}^{<}_j \cdot \vec{\mathcal{I}}_j &= \frac{1}{2\sqrt{2}}\left[A^{<}_j \left|-1\right\rangle \left|\uparrow\right\rangle_j \left\langle 0\right| \left\langle\downarrow\right|_j + \left(A^{<}_j\right)^* \left|0\right\rangle \left|\downarrow\right\rangle_j \left\langle -1\right| \left\langle\uparrow\right|_j\right] + A^{(j)}_{zz}\mathcal{S}_z\mathcal{I}^{(j)}_z, \\
\vec{\mathcal{S}} \cdot \mathbf{A}^{>}_j \cdot \vec{\mathcal{I}} &= \frac{1}{2\sqrt{2}}\left[A^{>}_j \left|-1\right\rangle \left|\downarrow\right\rangle_j \left\langle 0\right| \left\langle\uparrow\right|_j + \left(A^{>}_j\right)^* \left|0\right\rangle \left|\uparrow\right\rangle_j \left\langle -1\right| \left\langle\downarrow\right|_j\right] + A^{(j)}_{zz}\mathcal{S}_z\mathcal{I}^{(j)}_z,
\end{aligned} \tag{4}$$

respectively, where $|0\rangle$, $|-1\rangle$ refer to the NV spins states, and

$$\begin{aligned}
A^{<}_j &= A^{(j)}_{xx} + A^{(j)}_{yy}, \\
A^{>}_j &= A^{(j)}_{xx} - A^{(j)}_{yy} + 2iA^{(j)}_{xy}.
\end{aligned} \tag{5}$$

As these two transitions are spectrally distinct, from this point forward, we will simply refer to the transverse hyperfine coupling between the NV spin and spin j as A_j, where it is understood that its particular functional definition ($A^{<}_k$ or $A^{>}_k$) depends on the choice of resonant field strength, $B_<$ or $B_>$.

For a radial separation distance R_j and polar angle Θ_j, the effective transverse coupling rates between the NV spin and environmental spin j are given by

$$\begin{aligned} A_j^2 &= \left(A_{xx}^{(j)} \pm A_{yy}^{(k)}\right)^2 + \left(A_{xy}^{(j)} \mp A_{yx}^{(j)}\right)^2 \\ &= \frac{a^2}{R_j^6}\left[1 \pm 1 - 3\sin^2\left(\Theta_j\right)\right]^2, \end{aligned} \tag{6}$$

for magnetic fields of $B_<$, and $B_>$, respectively, corresponding to resonant transitions either side of the NV GSLAC.

For the $B_0 \sim 1000\,\mathrm{G}$ magnetic field strengths studied in this work, the Zeeman energies of the nuclear spins far exceed their mutual nuclear dipole couplings, hence any spin flip-flop dynamics they exhibit must be magnetisation conserving, and we may apply the secular approximation to the Hamiltonian describing their mutual dipole-dipole interaction

$$\vec{\mathcal{I}}_j \cdot \mathbf{B}_{jk} \cdot \vec{\mathcal{I}}_k = \sum_{k>j} B_{jk}\left(\mathcal{I}_x^{(j)}\mathcal{I}_x^{(k)} + \mathcal{I}_y^{(j)}\mathcal{I}_y^{(k)} - 2\mathcal{I}_z^{(j)}\mathcal{I}_z^{(k)}\right), \tag{7}$$

where

$$B_{jk} = \frac{b}{r_{jk}^3}\left[1 - 3\cos^2\left(\theta_{jk}\right)\right], \tag{8}$$

and r_{jk} and θ_{jk} are the separation distance and polar angle between spins j and k.

Discussion of competing time and length scales

^{13}C Environment

For the purposes of discussion of the relative strengths of the two transitions, it is useful to average over the angular components of Eq. 6, giving for before or after the GSLAC, respectively

$$\left\langle A_j^2 \right\rangle = \frac{4}{5}\frac{a^2}{R_j^6} \text{ or } \frac{24}{5}\frac{a^2}{R_j^6}, \tag{9}$$

where

$$a = \frac{\mu_0}{4\pi}\hbar\gamma_{\mathrm{NV}}\gamma_n. \tag{10}$$

Determination of the total hyperfine field strength then proceeds via summation over R_j, where we now index R_j according to the expected distance from the NV to its jth nearest ^{13}C spin; the distribution of which is given by Supplementary Ref. [2]

$$F\left(R_j\right) = \frac{4\pi n_{\mathrm{t}} R_j^2}{(j-1)!}\left(\frac{4\pi n_{\mathrm{t}} R_j^3}{3}\right)^{j-1} \exp\left(\frac{4\pi n_{\mathrm{t}} R_j^3}{3}\right),$$

where $n_{\mathrm{t}} = 1.95\,\mathrm{nm}^{-3}$ is the density of ^{13}C nuclei in diamond. Using this result in Eq. 9, we find

$$\begin{aligned} A_< &\approx 6.24 a n_{\mathrm{t}} \\ &= 1.43 \times 10^6 \mathrm{rad\,s}^{-1} \\ &= 228\,\mathrm{kHz} \end{aligned} \tag{11}$$

$$\begin{aligned} A_> &\approx 15.28 a n_{\mathrm{t}} \\ &= 3.51 \times 10^6 \mathrm{rad\,s}^{-1} \\ &= 559\,\mathrm{kHz}. \end{aligned} \tag{12}$$

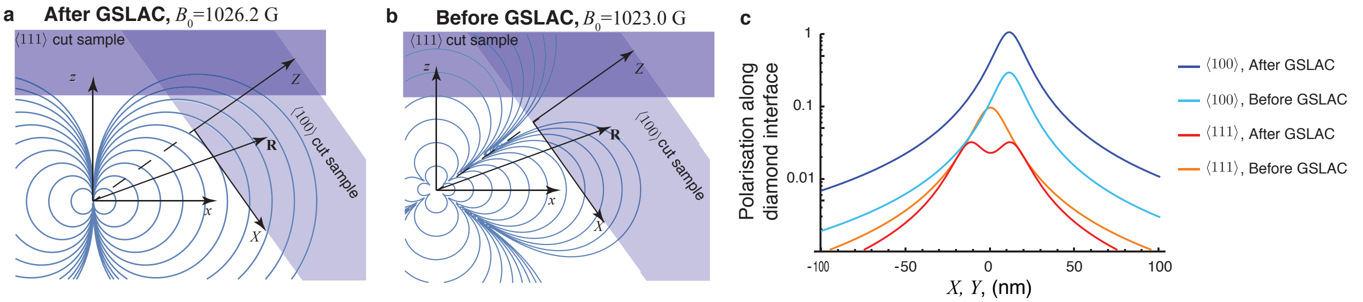

Supplementary Figure 2. **Coupling before and after the GSLAC comparison.** Contours of constant coupling for after (**a**) and before (**b**) the GSLAC with ⟨111⟩ and ⟨100⟩ surfaces shown in purple. **c** Polarisation profile at the diamond surface for all four possible scenarios, considering an NV located 10 nm below the surface and polarising a bath of PMMA spins.

^1H Environment

For the ^{1}H bath in PMMA, the NV-environment separation distance (approximately 10 nm) is much larger than the separation distances between adjacent ^{1}H spins. As such, the total hyperfine field strength may be evaluated via a standard integral over the effectively continuous, semi-infinite PMMA slab.

Due to the avoided crossing in NV spin level structure before the GSLAC (see Supplementary Fig. 1), the only resonance point between the NV spin and ^{1}H spins occurs after the GSLAC at $B_> = 1026.2\,\mathrm{G}$. In addition, the angular dependence of the interaction (see Eq. 6) must be considered when choosing the NV orientation relative to the surface. For instance, an NV normal to the surface of a ⟨111⟩ cut crystal, which is optimal as far as photon collection is concerned, would place the nearest ^{1}H spins in the node of the hyperfine coupling (see Supplementary Fig. 2). Here we use an NV in a standard ⟨100⟩ cut diamond crystal, which gives a relatively good coupling. As such, we transform the original coordinate system, $\mathbf{R} = (x, y, z)$ to the new coordinate system, $\mathbf{R}' = (X, Y, Z)$, defined by

$$\begin{aligned} x &= X\cos(\alpha) - Z\sin(\alpha) \\ y &= Y \\ z &= X\sin(\alpha) + Z\cos(\alpha), \end{aligned} \tag{13}$$

where $\alpha = \arccos\left(1/\sqrt{3}\right) \approx 54.7°$. The hyperfine integral then proceeds as

$$\begin{aligned} A_0^2 &= n_\mathrm{t}\int_h^\infty\int_{-\infty}^\infty\int_{-\infty}^\infty A^2\left(\mathbf{R}'\right)\,\mathrm{d}X\,\mathrm{d}Y\,\mathrm{d}Z \\ &= \frac{19\pi}{24}\frac{a^2 n_\mathrm{t}}{h^3} \end{aligned} \tag{14}$$

where $n_\mathrm{t} = 56\,\mathrm{nm}^{-3}$ is the density of ^{1}H spins in PMMA, and h is the depth of the NV below the diamond-PMMA interface (measured to be approximately 10 nm in this work).

Theoretical description of CRIP protocol in the absence of NV spin dephasing

In this section, we solve for the evolution of a number of special cases of Eq. **??** in order to motivate and develop a general continuum description for an environment containing an arbitrary number of spins.

Single hyperfine coupled spin

Assuming a starting density matrix of

$$\rho(0) = \left(|0\rangle\langle 0|\right)_\mathrm{NV} \otimes \left(p\,|\uparrow\rangle\langle\uparrow| + (1-p)\,|\downarrow\rangle\langle\downarrow|\right)_\mathrm{n}, \tag{15}$$

where p denotes the probability of the target spin being in its $|\uparrow\rangle$ state, the probability of finding the target spin in its $|\uparrow\rangle$ state at some later time, t, is given by

$$p(t) = p_1(0)\left[1 - \frac{A^2}{A^2+2\delta^2}\sin^2\left(\frac{t\sqrt{A^2+2\delta^2}}{2\sqrt{2}}\right)\right], \tag{16}$$

where $\delta = \omega_{\rm NV} - \omega_{\rm n}$ is the detuning between the transition frequencies of the NV and the target spin. By tuning the magnetic field such that $\delta = 0$, and choosing the evolution time to facilitate the maximum possible magnetisation exchange, $t = \frac{\sqrt{2}\pi}{A} \equiv \tau$, the probability of finding the spin in its up state becomes $p(\tau) = 0$, indicating that the polarisation of the NV has been transferred to the target spin. In the following, we will take $\delta = 0$ for simplicity. The effect of quasistatic magnetic noise, which amounts to a fluctuating detuning, will be be incorporated in the continuum description by introducting the NV dephasing rate, Γ_2.

Two independent hyperfine coupled spins

In the case of two target spins coupled to the NV, complete polarisation cannot be achieved within a single interaction. Assuming an initial state of

$$\rho(0) = \big(|0\rangle\langle 0|\big)_{\rm NV} \otimes \Big(p_1|\uparrow\rangle\langle\uparrow| + (1-p_1)|\downarrow\rangle\langle\downarrow|\Big)_{\rm t_1} \otimes \Big(p_2|\uparrow\rangle\langle\uparrow| + (1-p_2)|\downarrow\rangle\langle\downarrow|\Big)_{\rm t_2}, \tag{17}$$

the time-dependent $|0\rangle$ state population of the NV is

$$p_{\rm NV} = 1 - \frac{p_1A_1^2 + p_2A_2^2}{A_1^2+A_2^2+2\delta^2}\sin^2\left(t\frac{\sqrt{A_1^2+A_2^2+2\delta^2}}{2\sqrt{2}}\right). \tag{18}$$

Therefore, the maximum possible contrast corresponds to the minimum of $p_{\rm NV}$, given by

$$p_{\rm NV}^{\rm min} = 1 - \frac{p_1A_1^2+p_2A_2^2}{A_1^2+A_2^2}, \tag{19}$$

when $\delta = 0$ and

$$\begin{aligned} t &= \tau \\ &= \pi\sqrt{\frac{2}{A_1^2+A_2^2}}. \end{aligned} \tag{20}$$

Substituting τ into the expressions for p_1 and p_2, we find the change in populations to be

$$\begin{aligned} \Delta p_1 &= -p_1\frac{A_1^2}{A_1^2+A_2^2} - \big(p_1-p_2\big)\frac{A_1^2A_2^2}{(A_1^2+A_2^2)^2} \\ \Delta p_2 &= -p_2\frac{A_2^2}{A_1^2+A_2^2} - \big(p_2-p_1\big)\frac{A_1^2A_2^2}{(A_1^2+A_2^2)^2} \end{aligned} \tag{21}$$

The terms proportional to $p_1 - p_2$ are mixing terms that facilitate faster polarisation of the farthest spin due to the back action of the NV spin in response to the closest spin.

Two dipole coupled spins

We now add an additional magnetic dipole-dipole interaction between the two spins. In general, this system does not exhibit a closed-form solution, however, the timescale of the dipole coupling between the two environmental spins, $\tau_B \sim 1/B_{12}$, is much longer than that of the hyperfine dynamics, $\tau_A \sim 1/A$. Hence, we may expand the dipole-dipole dynamics for times $t \ll 1/B_{12}$ to give

$$\begin{aligned} \Delta p_1 &= -p_1\frac{A_1^2}{A_1^2+A_2^2} - \big(p_1-p_2\big)\frac{A_1^2A_2^2}{(A_1^2+A_2^2)^2} - 2\big(p_1-p_2\big)\frac{B_{12}^2}{A_1^2+A_2^2} \\ \Delta p_2 &= -p_2\frac{A_2^2}{A_1^2+A_2^2} - \big(p_2-p_1\big)\frac{A_1^2A_2^2}{(A_1^2+A_2^2)^2} - 2\big(p_2-p_1\big)\frac{B_{12}^2}{A_1^2+A_2^2}. \end{aligned} \tag{22}$$

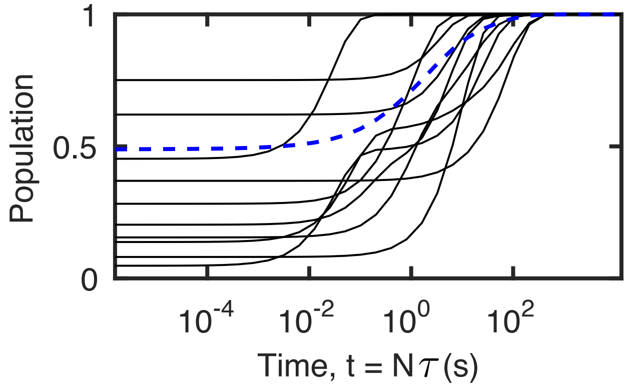

Supplementary Figure 3. Simulation of the naturally occurring (1.1% abundant) ^{13}C nuclear spin bath in the diamond crystal using 1000 randomly initialised ^{13}C spins. Solid lines represent the polarisation of 10 spins randomly chosen from the bath, the dashed line represents the average $|\uparrow\rangle$ state probability (i.e. polarisation) of all 1000 ^{13}C spins with interaction time $\tau = 3.8\,\mu$s.

For cases where both spins exist outside the strongly hyperfine coupled core surrounding the NV, we have that the two spins will thermalise on the timescale of their dipole coupling, τ_B. Hence, for outside the core on timescales of τ, we have

$$\begin{aligned}\Delta p_1 &= -p_1\frac{A_1^2}{A_1^2+A_2^2} - \left(p_1-p_2\right)\frac{A_1^2A_2^2}{(A_1^2+A_2^2)^2} - \frac{|B_{12}|}{2}\left(p_1-p_2\right)\tau \\ \Delta p_2 &= -p_2\frac{A_2^2}{A_1^2+A_2^2} - \left(p_2-p_1\right)\frac{A_1^2A_2^2}{(A_1^2+A_2^2)^2} - \frac{|B_{12}|}{2}\left(p_2-p_1\right)\tau.\end{aligned} \tag{23}$$

General case and continuum description

1. General case and continuum description

For the case of $N_{\rm S}$ spins, each having hyperfine coupling A_j to the NV, mutual dipole couplings B_{jk}, and initial state,

$$\rho(0) = \left(|0\rangle\langle 0|\right)_{\rm NV}\bigotimes_j\left(p_j\,|\uparrow\rangle\langle\uparrow| + (1-p_j)\,|\downarrow\rangle\langle\downarrow|\right)_{\rm t_j}, \tag{24}$$

the population of the NV $|0\rangle$ state is at a minimum when $t=\tau=\frac{\sqrt{2}\pi}{A_0}$, and is given by

$$p_{\rm NV}(\tau) = 1 - \sum_j \frac{A_j^2}{A_0^2}p_j(0), \tag{25}$$

where $A_0^2 \equiv \sum_j A_j^2$ is the total hyperfine coupling field.

The change in probability of spin j is given by

$$\Delta p_j = -p_j\frac{A_j^2}{A_0^2} - \sum_k\left(p_j-p_k\right)\left(\frac{\tau}{2}\,|B_{12}| + \frac{C_{jk}^2}{A_0^2}\right), \tag{26}$$

where B_{jk} is the magnetic dipole coupling between spins j and k, and $C_{jk}^2 \equiv A_j^2A_k^2/A^2$ is the effective hyperfine-mediated diffusion strength. For approximately $N_{\rm S}\sim 10^4$ spins, the evolution of discrete spins in this system may be solved numerically, an example of which is given in Fig. 3.

Spins residing in the hyperfine core are characterised by having a stronger coupling to the NV than to all other spins in the environment, i.e. $A_j^2 > \sum_k B_{jk}^2$. Typically we are interested in total times $t \gg \tau$ (hours vs μs, respectively) for which the polarisation region has far exceeded the hyperfine core, as a result of dipole-mediated flip-flops between the polarised core and the unpolarised outer region. As such, we are not interested in the relatively fast dynamics

associated with intra-core diffusion, and instead only concern ourselves with the rate at which the NV polarises spin in the core (A_k), and the rate at which this effect is communicated to the rest of the bath B_{jk}. We thus take $C_{jk} \sim 0$.

In modelling these systems, we typically want to consider regions of at least 200 nm in size, which means of order $N_{\mathrm{S}} \sim 10^{10}$ spins and $\sim 10^{20}$ couplings for the case of PMMA, thereby making modelling of discrete spin states computationally unfeasible. We therefore map Eq. 26 to a temporally and spatially continuous description via the following:

a. Time-dependent probability field – As the regions of polarisation considered in this work are larger than the hyperfine core of the environment, which itself is comprised of the order of 10^4 spins, instead of considering the time-dependent populations of discrete spins, we instead consider the time-dependent probability field of the environmental spins having an average density, n_{t},

$$p_j \mapsto p\left(\mathbf{R}, t\right). \tag{27}$$

In essence, $p\left(\mathbf{R}, t\right)$ is the average probability of spins in an infinitesimal volume at position $\mathbf{R}$ from the NV being in state $|\uparrow\rangle$ at time t.

b. Probability field dynamics – In the discrete description, the probability of finding spin j in its $|\uparrow\rangle$ state is monitored at discrete multiples of the optimal dark time, τ. Ignoring probability diffusion, this would behave as a geometric series. As we are interested in times $t \gg \tau$, we map changes in p_j over time to

$$\Delta p_j \mapsto \tau \frac{\partial}{\partial t} p\left(\mathbf{R}, t\right) \tag{28}$$

c. Hyperfine coupling field – As with the population field, we also map the discrete hyperfine couplings to a continuous field whose strength is determined by its position relative to the NV, $\mathbf{R}$.

$$A_j \mapsto A\left(\mathbf{R}\right). \tag{29}$$

d. Total hyperfine coupling strength – The summation over all hyperfine couplings is mapped to an integral,

$$A_0^2 \mapsto n_{\mathrm{t}} \int A^2\left(\mathbf{R}\right) \, \mathrm{d}^3\mathbf{R}. \tag{30}$$

e. Probability diffusion – We put $p_j \mapsto p\left(\mathbf{R}, t\right)$ and $p_k \mapsto p\left(\mathbf{R} + \mathbf{r}, t\right)$, and discretise the Laplacian operator to get

$$\sum_k \left(p_j - p_k\right) B_{jk} \mapsto \langle r\rangle^2 \sqrt{\langle B^2\rangle} \nabla^2 p\left(\mathbf{R}, t\right). \tag{31}$$

f. Additional sources of relaxation – To account for additional sources of environmental spin-lattice relaxation, where applicable, we add an additional probability sink in which any probability outside equilibrium decays at a rate $\Gamma_{\mathrm{SL}} = \frac{1}{T_{\mathrm{SL}}}$, whose rate is given by

$$R_{\mathrm{SL}} = -\Gamma_{\mathrm{SL}} \left(p\left(\mathbf{R}, t\right) - \frac{1}{2}\right). \tag{32}$$

The differential equation describing the evolution of this system is thus given by

$$\frac{\partial}{\partial t} p\left(\mathbf{R}, t\right) = -u(\mathbf{R}) p\left(\mathbf{R}, t\right) + \beta \nabla^2 p(\mathbf{R}, t) - \Gamma_{\mathrm{SL}} \left(p\left(\mathbf{R}, t\right) - \frac{1}{2}\right), \tag{33}$$

where

$$u(\mathbf{R}) = \frac{A^2(\mathbf{R})}{\tau A_0^2} \tag{34}$$

is the effective source, or cooling, coefficient at position $\mathbf{R}$ relative to the NV, and

$$\beta = \frac{1}{2} \langle r\rangle^2 \sqrt{\langle B^2\rangle} \tag{35}$$

is the effective probability diffusion coefficient related to the intra-bath interactions B_{jk}. Recasting Eq. 33 in terms of the polarisation $P(\mathbf{R}, t) = 1 - 2p(\mathbf{R}, t)$, we obtain Equation (1) of the main text.

Quantity	^{13}C, $B_0 = 1023.9$	^{13}C, $B_0 = 1024.9$	^{1}H, $B_0 = 1026.2\,\mathrm{G}$
n_{t}	$1.95\,\mathrm{nm}^{-3}$	$1.95\,\mathrm{nm}^{-3}$	$56\,\mathrm{nm}^{-3}$
$\sqrt{A_0^2}$	$1.43 \times 10^6\,\mathrm{rad\,s}^{-1}$	$3.51 \times 10^6\,\mathrm{rad\,s}^{-1}$	$176 \times 10^3\,\mathrm{rad\,s}^{-1}$
$u\left(\mathbf{R}\right)$	$27 \times 10^3\,\mathrm{s}^{-1}\left[1 - 3\cos^2(\Theta)\right]^2 R^{-6}$	$101 \times 10^3\,\mathrm{s}^{-1}\sin^4(\Theta)R^{-6}$	$714 \times 10^3\,\mathrm{s}^{-1}\frac{\left(2h^2+2\sqrt{2}hX+4hZ+X^2+2\sqrt{2}XZ+3Y^2+2Z^2\right)^2}{\left((h+Z)^2+X^2+Y^2\right)^5}$
$\sqrt{\langle B^2\rangle}$	$460\,\mathrm{rad\,s}^{-1}$	$460\,\mathrm{rad\,s}^{-1}$	$40 \times 10^3\,\mathrm{rad\,s}^{-1}$
β	$8.22 \times 10^{-2}\,\mathrm{nm}^2\mathrm{s}^{-1}$	$3.35 \times 10^{-2}\,\mathrm{nm}^2\mathrm{s}^{-1}$	$93.53\,\mathrm{nm}^2\mathrm{s}^{-1}$
Γ_{SL}	$\ll A_0$	$\ll A_0$	$1\ \mathrm{s}^{-1}$
Γ_2	$2 \times 10^5\,\mathrm{rad\,s}^{-1} \ll A_0$	$2 \times 10^5\,\mathrm{rad\,s}^{-1} \ll A_0$	$1.5 \times 10^6\,\mathrm{rad\,s}^{-1}$

Supplementary Table 1. Summary of parameters used to describe the different scenarios examined in this work: the intrinsic ^{13}C bath in diamond (first two column, for before and after GSLAC resonances, respectively), and the ^{1}H bath formed by a PMMA slab on diamond with a 10-nm-deep NV (third column).

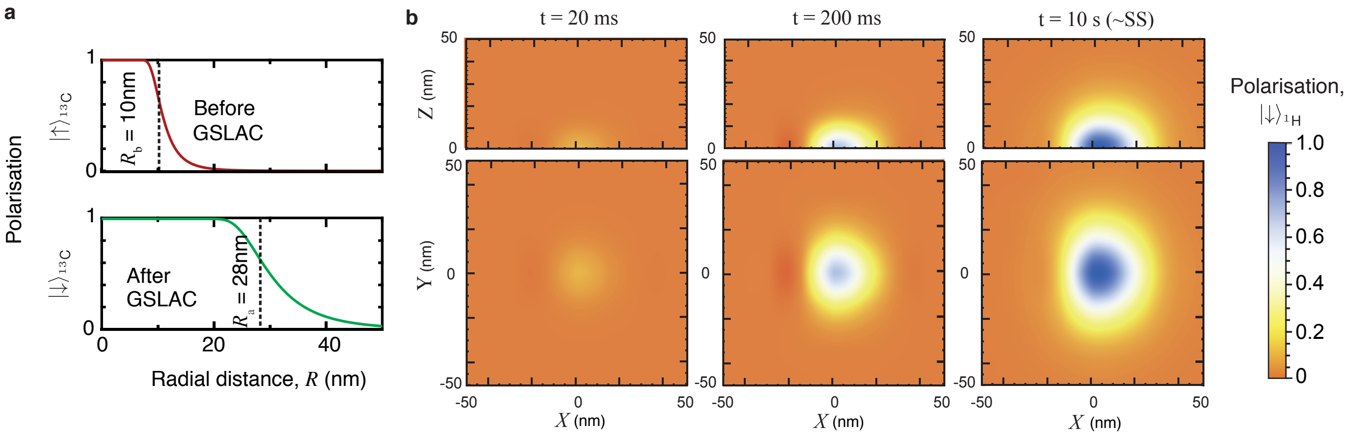

Supplementary Figure 4. **Spatial extent of polarisation in the ^{13}C spin bath.** **a** Theoretical radial polarisation profiles for ^{13}C resonances before (red) and after (green) the GSLAC constructed using their respective experimentally measured longitudinal relaxation rates after approximately 5 hours of polarisation. **b** Theoretical ^{1}H polarisation distributions in the $X - Z$ and $X - Y$ planes of the PMMA layer for total polarisation times of $t = 20\,\mathrm{ms}$, $200\,\mathrm{ms}$, and $10\,\mathrm{s}$ (steady state, SS). The PMMA-diamond interface defines $Z = 0$.

For cases in which the dephasing rate of the NV spin, Γ_2, is greater than the hyperfine coupling to the desired target, i.e. $\Gamma_2 \gg A_0$, the population of $|0\rangle$ state of the NV spin will exhibit an exponential time dependence. As such, for this regime we instead make the replacement

$$u\left(\mathbf{R}\right) = \frac{A^2(\mathbf{R})}{2\Gamma_2}, \tag{36}$$

where the details of this process are discussed in the following section.

Experimentally, Γ_2 is obtained via the T_1-relaxometry spectrum (with the interleaved sequence), since Γ_2 defines the width of the cross-relaxation resonances [2]. We note that Γ_2 is expected to decrease upon polarisation of the ^{13}C bath. However, the effect is relatively small (see section Supplementary Note 2), and as such in the calculations shown in main text Fig. 2 we kept Γ_2 constant and equal to the off-resonance value (i.e. unpolarised case). For the ^{1}H case, the dephasing is dominated by surface effects, and is therefore unaffected by the ^{1}H polarisation.

A summary the parameters associated with the three systems studied is given in Table 1, detailing: the diamond ^{13}C bath before the GSLAC (Supplementary Fig. 8) and after the GSLAC (main text Fig. 2), and the PMMA ^{1}H bath after the GSLAC (main text Fig. 3).

The reconstructed radial profiles for the ^{13}C polarisation in the main text are shown in Supplementary Fig. 4**a** for both before and after the GSLAC. In a similar fashion the PMMA polarisation can be reconstructed. 2D slices of the polarisation extent in the PMMA for different polarisation times are shown in Supplementary Fig. 4**b**.

Theoretical description of CRIP protocol in the presence of NV spin dephasing

The above development outlines the behaviour of the spin bath under CRIP for cases in which NV spin dephasing is not appreciable over the interaction time, τ. In what follows, we discuss how this behaviour is modified in the opposite regime. To motivate the discussion, we recall the semi-classical result from Supplementary Ref. [2], which gives the response of an NV spin prepared in the $|0\rangle$ state to an effective hyperfine field of magnitude A, and transverse spin relaxation (dephasing) rate, Γ_2,

$$p_{\rm NV} = \frac{1}{2} + \frac{1}{2}\exp\left(-\Gamma_{\rm CR} t\right), \tag{37}$$

where

$$\Gamma_{\rm CR} = \frac{A^2\Gamma_2}{2\Gamma_2^2 + 2\left(\omega_{\rm NV} - \omega_{\rm n}\right)^2}. \tag{38}$$

In the semi-classical sense, A refers to the coupling of the effective magnetic field felt to the transverse components of the NV spin due to all target spins the environment. The magnitude of this field depends on the orientation of the individual spins, and is related to the population of finding them in the $|\uparrow\rangle$ state, p_j, and the hyperfine coupling of the NV spin to spin j, A_j, via

$$\begin{aligned} A^2 &\mapsto A_p^2 \\ &= \sum_j p_j A_j^2. \end{aligned} \tag{39}$$

Naturally a bath in which every spin is polarised in its $|\downarrow\rangle$ state will not be able to flip the NV spin, leading to no relaxation effect; however a bath in which all spins are in the $|\uparrow\rangle$ state will have the greatest effect. For cases in which the baths are sufficiently large that the spins effectively comprise a continuum, this becomes

$$\begin{aligned} A_p^2 &\mapsto n_{\rm t}\int p\left(\mathbf{R}\right) A^2\left(\mathbf{R}\right)\,{\rm d}^3\mathbf{R}, \\ &= \frac{n_{\rm t}}{2}\int \left[1 - P\left(\mathbf{R}\right)\right] A^2\left(\mathbf{R}\right)\,{\rm d}^3\mathbf{R}, \end{aligned} \tag{40}$$

which is the case for both the native ^{13}C and PMMA ^{1}H baths considered in this work. In the case of the latter, the distance between the NV and the nearest target spin is 10 nm, whereas their average separation is only a few angstrom. Whilst this is not the case in general for the former, the target spins close to the NV polarise so quickly under CRIP that the effective standoff between the NV and the unpolarised spins in the target bath very quickly becomes larger than the separation between target spins. In what follows, we derive a quantum mechanical description of the NV spin relaxation, and show that it agrees with the classical result for the cases considered in this work. As with the no dephasing case, we consider examples of only a few spins, and show how this generalises to large target baths.

Single target spin

For the case of a single spin, we follow Supplementary Ref. [2] and consider the evolution of the density matrix as described by

$$\frac{{\rm d}\rho}{{\rm d}t} = -i\left[H, \rho\right] + \sqrt{\frac{\Gamma_2}{2}}\left(\sigma_z\rho\sigma_z - \rho\right), \tag{41}$$

where $\sigma_z = |0\rangle\langle 0| - |-1\rangle\langle -1|$ operates on the NV spin; subject to initial state $\rho_{11} = p_1$; $\rho_{22} = 1 - p_1$, with all other $\rho_{jk} = 0$. This gives rise to the following differential equations,

$$\begin{aligned} \frac{{\rm d}^3}{{\rm d}t^3}\rho_{11} + 2\Gamma_2\frac{{\rm d}^2}{{\rm d}t^2}\rho_{11} + \left(\frac{A_1^2}{2} + \delta^2 + \Gamma_2^2\right)\frac{{\rm d}}{{\rm d}t}\rho_{11} + \frac{A_1^2}{2}\Gamma_2\rho_{11} + \frac{A_1^2}{4}\Gamma_2 p_1 = 0, \\ \frac{{\rm d}}{{\rm d}t}\rho_{22} = 0, \end{aligned} \tag{42}$$

which, in the limit of $\Gamma_2^2 \gg A_1^2$, results in an NV $|0\rangle$ population of

$$p_{\rm NV} = 1 + \frac{1}{2}\left[\exp\left(-\Gamma_{\rm CR} t\right) - 1\right] p_1, \tag{43}$$

where, as in the classical case,

$$\Gamma_{\mathrm{CR}} = \frac{A_{\mathrm{p}}^2\Gamma_2}{2\Gamma_2^2 + 2\left(\omega_{\mathrm{NV}} - \omega_{\mathrm{n}}\right)^2}. \tag{44}$$

Two target spins

For the case of a two spin target bath, there are now three states that couple to the $|0\rangle$ state of the NV spin: $|\uparrow\rangle|\uparrow\rangle$, $|\uparrow\rangle|\downarrow\rangle$, and $|\downarrow\rangle|\uparrow\rangle$. Following the same process as for the single spin case, but for these three transitions, we have

$$p_{\mathrm{NV}} = \frac{1}{2}p_1p_2\left(e^{-\Gamma_{\mathrm{CR}}^{\uparrow\uparrow}t}+1\right) + \frac{1}{2}p_1\left(1-p_2\right)\left(e^{-\Gamma_{\mathrm{CR}}^{\uparrow\downarrow}t}+1\right) + \frac{1}{2}\left(1-p_1\right)p_2\left(e^{-\Gamma_{\mathrm{CR}}^{\downarrow\uparrow}t}+1\right) + \left(1-p_1\right)\left(1-p_2\right), \tag{45}$$

where $p_{1,2}$ is the probability of spin 1,2 being in its $|\uparrow\rangle$ state. From this, we see that the populations of the four possible basis states decay with rates of

$$\Gamma_{\mathrm{CR}}^{\uparrow\uparrow} = \frac{\left(A_1^2 + A_2^2\right)\Gamma_2}{2\Gamma_2^2 + 2\left(\omega_{\mathrm{NV}} - \omega_{\mathrm{n}}\right)^2}, \tag{46}$$

$$\Gamma_{\mathrm{CR}}^{\uparrow\downarrow} = \frac{A_1^2\Gamma_2}{2\Gamma_2^2 + 2\left(\omega_{\mathrm{NV}} - \omega_{\mathrm{n}}\right)^2}, \tag{47}$$

$$\Gamma_{\mathrm{CR}}^{\downarrow\uparrow} = \frac{A_2^2\Gamma_2}{2\Gamma_2^2 + 2\left(\omega_{\mathrm{NV}} - \omega_{\mathrm{n}}\right)^2}, \tag{48}$$

$$\Gamma_{\mathrm{CR}}^{\downarrow\downarrow} = 0, \tag{49}$$

respectively. Thus we see that the decay rate of the NV spin depends on the initial state of the target bath, and consequently, on the individual polarisations of the bath constituents.

General case

Extending this to the many spin case, we see that the NV $|0\rangle$ state population in response to the target being initialised in the product basis state $|\sigma_1\sigma_2\ldots\rangle$, where $\sigma_k = 1, 0$ corresponds to $|\uparrow\rangle, |\downarrow\rangle$ respectively, is given by

$$Q_{\mathrm{NV}}(\sigma_1,\sigma_2,\ldots) = \frac{1}{2}q(\sigma_1,\sigma_2,\ldots)\left[1 + \exp\left(-\Gamma_{\mathrm{CR}}(\sigma_1,\sigma_2,\ldots)t\right)\right], \tag{50}$$

where,

$$\Gamma_{\mathrm{CR}}(\sigma_1,\sigma_2,\ldots) = \frac{1}{2}\frac{\Gamma_2}{\Gamma_2^2+\delta^2}\sum_k \sigma_k A_k^2, \tag{51}$$

is the decay rate out of product state $|\sigma_1\sigma_2\ldots\rangle$, and $q(\sigma_1,\sigma_2,\ldots)$ is the initial probability of being in that state.

In order to relate this process to the individual spin probabilities, p_j, we note that for a random initial state, each spin has an associated initial density state given by

$$\rho_j(0) = p_j\left|\uparrow_j\right\rangle\left\langle\uparrow_j\right| + \left(1-p_j\right)\left|\downarrow_j\right\rangle\left\langle\downarrow_j\right|, \tag{52}$$

where the initial full target density state is $\rho(0) = \prod_k \rho_k(0)$. From this, we see that

$$q(\sigma_1,\sigma_2,\ldots) = \prod_j\left[1 - p_j + \left(2p_j - 1\right)\sigma_j\right]. \tag{53}$$

The full NV $|0\rangle$ state population is just the sum over all possible initial states associated with Eq. 50,

$$
\begin{aligned}
p_{\mathrm{NV}} &= \sum_{\sigma_1,\sigma_2,\ldots} Q_{\mathrm{NV}}(\sigma_1,\sigma_2,\ldots) \\
&= \frac{1}{2}\sum_{\sigma_1,\sigma_2,\ldots}\prod_j \left[1-p_j+(2p_j-1)\,\sigma_j\right]\left[1+\exp\left(-\frac{t}{2}\frac{\Gamma_2}{\Gamma_2^2+\delta^2}\sum_k \sigma_k A_k^2\right)\right] \\
&= \frac{1}{2}+\frac{1}{2}\sum_{\sigma_1,\sigma_2,\ldots}\prod_j \left[1-p_j+(2p_j-1)\,\sigma_j\right]\left[\exp\left(-\frac{t}{2}\frac{\Gamma_2}{\Gamma_2^2+\delta^2}\sigma_j A_j^2\right)\right] \\
&= \frac{1}{2}+\frac{1}{2}\sum_{\sigma_1,\sigma_2,\ldots}\prod_j \left[1-p_j+(2p_j-1)\,\sigma_j\right]\left[1+\sum_{n=1}^{\infty}\frac{1}{n!}\left(-\frac{t}{2}\frac{\Gamma_2}{\Gamma_2^2+\delta^2}\sigma_j A_j^2\right)^n\right] \\
&= \frac{1}{2}+\frac{1}{2}\sum_{\sigma_1,\sigma_2,\ldots}\prod_j \left[\left[1-p_j+(2p_j-1)\,\sigma_j\right]+\sigma_j p_j\sum_{n=1}^{\infty}\frac{1}{n!}\left(-\frac{t}{2}\frac{\Gamma_2}{\Gamma_2^2+\delta^2}A_j^2\right)^n\right] \\
&= \frac{1}{2}+\frac{1}{2}\sum_{\sigma_1,\sigma_2,\ldots}\prod_j \left[1-p_j-(1-p_j)\,\sigma_j+\sigma_j p_j+\sigma_j p_j\sum_{n=1}^{\infty}\frac{1}{n!}\left(-\frac{t}{2}\frac{\Gamma_2}{\Gamma_2^2+\delta^2}A_j^2\right)^n\right] \\
&= \frac{1}{2}+\frac{1}{2}\sum_{\sigma_1,\sigma_2,\ldots}\prod_j \left[1-p_j-(1-p_j)\,\sigma_j+\sigma_j p_j\exp\left(-\frac{t}{2}\frac{\Gamma_2}{\Gamma_2^2+\delta^2}A_j^2\right)\right] \\
&= \frac{1}{2}+\frac{1}{2}\sum_{\sigma_1,\sigma_2,\ldots}\prod_j \left[(1-p_j)\,(1-\sigma_j)+\sigma_j p_j\exp\left(-\frac{t}{2}\frac{\Gamma_2}{\Gamma_2^2+\delta^2}A_j^2\right)\right] \\
&= \frac{1}{2}+\frac{1}{2}\prod_j \left[1+p_j\exp\left(-\frac{t}{2}\frac{\Gamma_2}{\Gamma_2^2+\delta^2}A_j^2\right)-p_j\right] \\
&= \frac{1}{2}+\frac{1}{2}\exp\log\prod_j \left[1+p_j\exp\left(-\frac{t}{2}\frac{\Gamma_2}{\Gamma_2^2+\delta^2}A_j^2\right)-p_j\right] \\
&= \frac{1}{2}+\frac{1}{2}\exp\sum_j\log \left[1+p_j\exp\left(-\frac{t}{2}\frac{\Gamma_2}{\Gamma_2^2+\delta^2}A_j^2\right)-p_j\right] \qquad (54)
\end{aligned}
$$

For cases in which the NV-target standoff is much larger than the average distance between the target spins, many spins will couple to the NV spin simultaneously and with similar strength. The contribution from each individual spin $\left(\sim A_j^2/2\Gamma_2\right)$ will therefore be necessarily small as compared with the total NV spin relaxation rate $\left(\sim \sum_j A_j^2/2\Gamma_2\right)$, meaning that we may formally expand both the exponential term and the logarithm above to first order in $A_j^2/\sum A_j^2$, giving

$$
\begin{aligned}
p_{\mathrm{NV}} &\approx \frac{1}{2}+\frac{1}{2}\exp\left[-\frac{t}{2}\frac{\Gamma_2}{\Gamma_2^2+\delta^2}\sum_j p_j A_j^2\right] \\
&\mapsto \frac{1}{2}+\frac{1}{2}\exp\left[-\frac{t}{2}\frac{\Gamma_2}{\Gamma_2^2+\delta^2}\int n_t p\left(\mathbf{R}\right)A^2\left(\mathbf{R}\right)\,\mathrm{d}^3\mathbf{R}\right], \qquad (55)
\end{aligned}
$$

in agreement with the classical result (Eqs. 37-40).

Scale-up strategy

Having analysed the CRIP protocol above for the case of a single NV spin, we now discuss how this technique may be scaled up for the purpose of polarising macroscopic quantities of arbitrary nuclear spin labels for clinical applications such as MRI imaging.

Polarisation rates

From the analysis above, the maximum rate (spins/s) at which polarisation is delivered to the target spin ensemble from a single NV is equal to the effective hyperfine field,

$$\begin{aligned} R_{\mathrm{single}} &= A \\ &= \frac{1}{2} a \sqrt{\frac{n_{\mathrm{t}}}{h_{\mathrm{NV}}^{3}}} \sqrt{\frac{19\pi}{6}} \end{aligned} \tag{56}$$

where n_{t} is the numerical density of the target spins, h_{NV} is the distance of the NV from the diamond/target interface; and $a = \frac{\mu_0}{4\pi}\gamma_{\mathrm{NV}} g_{\mathrm{T}} \mu_{\mathrm{N}}$, where g_{T} is the g-factor of the target species, and μ_{N} is the nuclear magneton. For a planar NV density within the diamond substrate of σ, the total polarisation delivery rate is

$$R = \sigma \times \mathrm{Area} \times R_{\mathrm{single}}. \tag{57}$$

Time-dependent polarisation

Assuming that the spatial diffusion of target spins is sufficiently fast to ensure perfect mixing (i.e. uniform polarisation throughout the cell on timescales of $\sim 1/A$), the polarisation of the target spins is governed by

$$\frac{\mathrm{d}P}{\mathrm{d}T} = R\,(1-P) - \frac{P}{T_{\mathrm{SL}}}, \tag{58}$$

where P is the average polarisation of the target spins, and T_{SL} is their spin-lattice relaxation time. Solution of this equation yields

$$P(T) = \frac{RT_{\mathrm{SL}}}{N + RT_{\mathrm{SL}}} \left[1 - \exp\left(-T \frac{N + RT_{\mathrm{SL}}}{NT_{\mathrm{SL}}}\right)\right], \tag{59}$$

where $N \equiv n_{\mathrm{t}} \times \mathrm{Area} \times h_{\mathrm{cell}}$ is the total number of target spins in the cell, and h_{cell} is the height of the cell.

Optimal geometry

From Eq. 59, we see that the steady-state polarisation and characteristic polarisation time are given by

$$P_{\mathrm{SS}} = \frac{RT_{\mathrm{SL}}}{N + RT_{\mathrm{SL}}} \tag{60}$$

$$\tau_P = \frac{NT_{\mathrm{SL}}}{N + RT_{\mathrm{SL}}} \tag{61}$$

respectively. It is clear that these expressions define a critical effective number of spins, $N_{\mathrm{crit}} \equiv RT_{\mathrm{SL}}$, that may be polarised before that polarisation is lost to spin-lattice relaxation effects. As such, any delivery system of spin-polarised contrast agents necessitates that $N_{\mathrm{crit}} \gg N$. As both of these quantities are proportional to the area of the NV array, we may rewrite this constraint in terms of the coupling parameters h_{NV} and cell height h_{cell} to give

$$h_{\mathrm{cell}} \ll h_{\mathrm{crit}}, \tag{62}$$

where

$$\begin{aligned} h_{\mathrm{crit}} &= \frac{a}{2} \sqrt{\frac{19\pi}{6}} T_{\mathrm{SL}} \sigma_{\mathrm{NV}} \sqrt{\frac{1}{n_{\mathrm{t}} h_{\mathrm{NV}}^{3}}} \\ &\approx 65\,\mathrm{nm\,s^{-1}} \times g_{\mathrm{T}} T_{\mathrm{SL}} \sqrt{N_{\mathrm{m}}}, \end{aligned} \tag{63}$$

where N_{m} is the number of target spins in the target molecule, and we have assumed a target concentration of 1 M. This allows us to define an effective length scale by which to characterise the suitability of candidate systems to this scheme.

Species	HEP	N-TMPA	H_2O
Nuclear g-factor, $g_{\rm T}$	1.405	0.5638	5.586
Number of target spins in molecule, $N_{\rm m}$	5	1	2
Gyromagnetic ratio, $\gamma_{\rm T}$ $\left(\mathrm{rad\,s^{-1}\,T^{-1}}\right)$	6.7×10^7	2.7×10^7	2.67×10^8
Spin-lattice relaxation time, $T_{\rm SL}$ (s)	100	600	3
Critical height, $h_{\rm crit}$ (µm)	20	22	3

Supplementary Table 2. Values used for the scaling up in the main text.

Volume delivery rate

From Eq. 59, the time required to achieve a desired polarisation, P, is

$$T_P = \frac{N T_{\rm SL}}{N + R T_{\rm SL}} \log\left(-\frac{R T_{\rm SL}}{NP + (P-1) R T_{\rm SL}}\right), \tag{64}$$

and the volume delivery rate of polarised contrast agent is

$$Q = \frac{d h_{\rm cell}}{T_P} \times \text{Area}, \tag{65}$$

where d is the dilution factor, typically of order 10^3 to reach mM concentrations. In the limit of $R T_{\rm SL} \gg N$, this becomes

$$\begin{aligned} Q &= \frac{dR}{n_{\rm t} \log\left(\dfrac{1}{1-P}\right)} \\ &= 1.03434\,\mathrm{\mu L\,s^{-1}} \times \frac{g_{\rm T}\sqrt{N_{\rm m}}}{\log\left(\dfrac{1}{1-P}\right)} \end{aligned} \tag{66}$$

Notice that $T_{\rm SL}$ does not enter into Eq. 66, as we have assumed $R T_{\rm SL} \gg N$, and hence $h_{\rm cell} \ll h_{\rm crit}$. Values of these quantities are given in Table 2 for various candidate contrast agents.

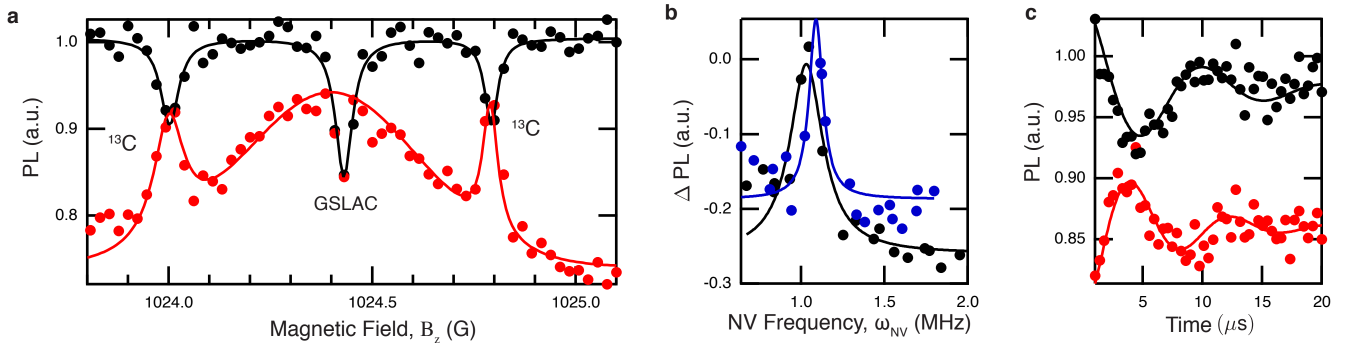

Supplementary Figure 5. **Cross-relaxation between a single NV and the ^{13}C bath. a** T_1-relaxometry spectrum performed around the GSLAC (middle feature) with two signals (outer features) corresponding to the ^{13}C resonances, with a wait time $\tau = 5\mu$s. The black and red lines ($|0\rangle$ and $|-1\rangle$) are obtained with a CRIP and CRIP^{-1} interleaved pulse sequence. The pulse sequence acts to prevent polarising the bath and allows the NV-^{13}C interaction to be measured. The solid line is a guide to the eye. **b** The difference between the $|-1\rangle$ and $|0\rangle$ state (ΔPL = PL($|-1\rangle$)− PL($|0\rangle$)) against the transition frequency measured in ODMR for before the GSLAC (black) and after (blue). **c** Short time spin dynamics using the same interleaved pulse sequence and varying the interaction time, τ. The measurement is taken on resonance with the ^{13}C bath (after GSLAC), revealing flip-flop interaction between the NV and the ^{13}C bath with a total hyperfine coupling of 250 ± 40 kHz.

Supplementary Note 2. ADDITIONAL EXPERIMENTAL INFORMATION

^{13}C detection and polarisation

Before proceeding with the polarisation the ^{13}C spin bath, the T_1-relaxometry spectrum of the ^{13}C bath was first obtained using the interleaved sequence, while scanning the magnetic field across the GSLAC. The full spectrum is shown in Supplementary Fig. 5**a** for the NV used in main text Fig. 2. The spectrum resolves three peaks, the two outer peaks correspond to the signal from the ^{13}C (before and after the GSLAC), the inner peak is an intrinsic feature of the GSLAC Supplementary Ref. [1]. The GSLAC feature of the $|0\rangle$ state (black, readout from the CRIP sequence) is narrower than that of the $|-1\rangle$ state (red, readout from the CRIP^{-1} sequence) due to experimental errors in implementing a π-flip in this region. This is caused by the rotating wave approximation breaking in this regime. This is because the driving field term in the Hamiltonian becomes similar in size to the quantisation axis itself. This needs to be considered for pulse experiments near the GSLAC as it can lead to artefacts in the measurements.

To elucidate the origin of the signal the PL difference (ΔPL) between the $|0\rangle$ and $|-1\rangle$ states versus the transition frequency ($\omega_{\rm NV}$) is shown in Supplementary Fig. 5**b**. The expected transition frequency, in the weakly coupled regime, is $\omega_C = \gamma_C B_z \approx 1.09$ MHz. There is a slight shift in the frequency measured which is possibly due to a hyperfine coupling between the NV and the nearest ^{13}C spins that remain slightly polarised. Measuring the short time dynamics of the NV spin on resonance shows a coherent evolution caused by the hyperfine coupling to the surrounding ^{13}C spins. The frequency of the oscillation corresponds to the sum of the total hyperfine coupling of the bath to the NV ($A_0^2 = \sum_k A_k^2$, see section S1) but will be dominated by the nearest ^{13}C. The damping is given by fluctuations in the spin configuration of the remaining bath spins, which causes the detuning, δ, to randomly vary. This is shown in Supplementary Fig. 5**c** and has an approximate coupling strength of 250 ±40 kHz.

The NV-bath coupling strength depends on the particular configuration of the bath around a given NV. We therefore expect a variability in the spectrum and degree of polarisation depending on the specific location of the ^{13}C spins in relation to the central NV spin. The ^{13}C polarisation has been tested over a variety of diamonds including deep NVs near surface NVs in bulk diamond, and NVs in micro-pillars. All of the different samples and NVs have shown the capability to polarise the ^{13}C spin bath provided that the transition is observable around the GSLAC, which requires the spin dephasing time of the NV, T_2^*, to be sufficiently long Supplementary Ref. [1]. The full CRIP and interleaved spectra (including the GSLAC feature) for the NV used in main text Fig. 2 are shown in Supplementary Fig. 6**a**. Also shown in Supplementary Fig. 6**b** are the spectra from another NV. The time dynamics for this NV on resonance with the ^{13}C bath is shown in Supplementary Fig. 6**c**, revealing a similar hyperfine coupling.

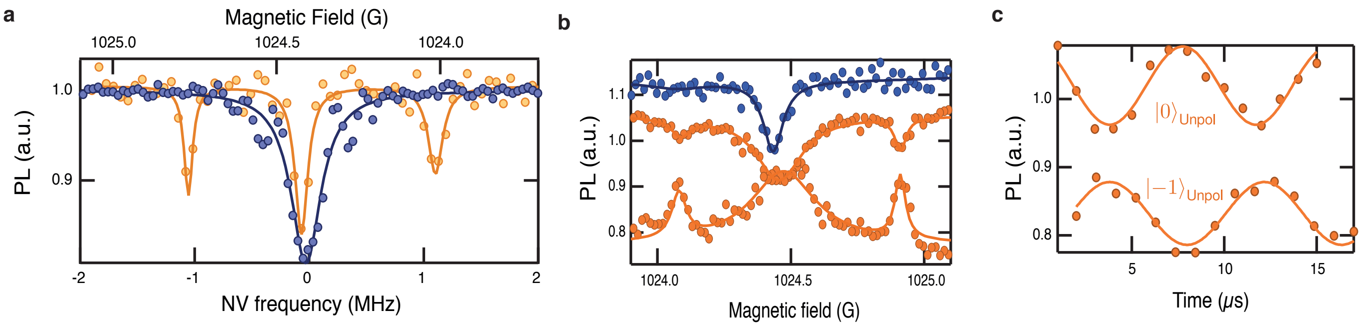

Supplementary Figure 6. **Polarising the ^{13}C bath. a** The ^{13}C spectrum (orange) obtained using the depolarisation sequence and the polarised spectrum (blue) using CRIP. This is the same NV as the one in main text Fig. 2. **b** Spectrum of ^{13}C around the GSLAC with polarisation (blue) and without polarisation (orange) on a different NV. With the interleaved sequence, both NV initialisation states are shown, $|0\rangle$ (upper orange) and $|-1\rangle$ (lower orange). **c** Time dynamics of the NV-bath interaction at the after-GSLAC ^{13}C resonance.

^{13}C polarisation dynamics

The polarisation dynamics was investigated for the NV used in main text Fig. 2, when on resonance with the ^{13}C bath (after the GSLAC). To this end, a series of $N = 30$ pulses was performed with a π pulse (i.e. CRIP^{-1}), which polarises the ^{13}C in the $|\uparrow\rangle$ state. This is followed by N pulses without the π pulse (i.e. CRIP) polarising the ^{13}C bath in the $|\downarrow\rangle$ state. The pulse sequence is shown in Supplementary Fig. 7**a**. The PL readout for each laser pulse as a function pulse number for three interaction times $\tau = 3, 5, 10\,\mu$s is shown in Supplementary Fig. 7**b**. The resulting polarisation curves were fitted with exponentials ($\mathrm{PL} = a\exp{(N/T_{\rm pol})} + y_0$) in order to determine the characteristic polarisation time. The result of the fits is shown in Supplementary Fig. 7**c** and shows a clear trend of decreasing the

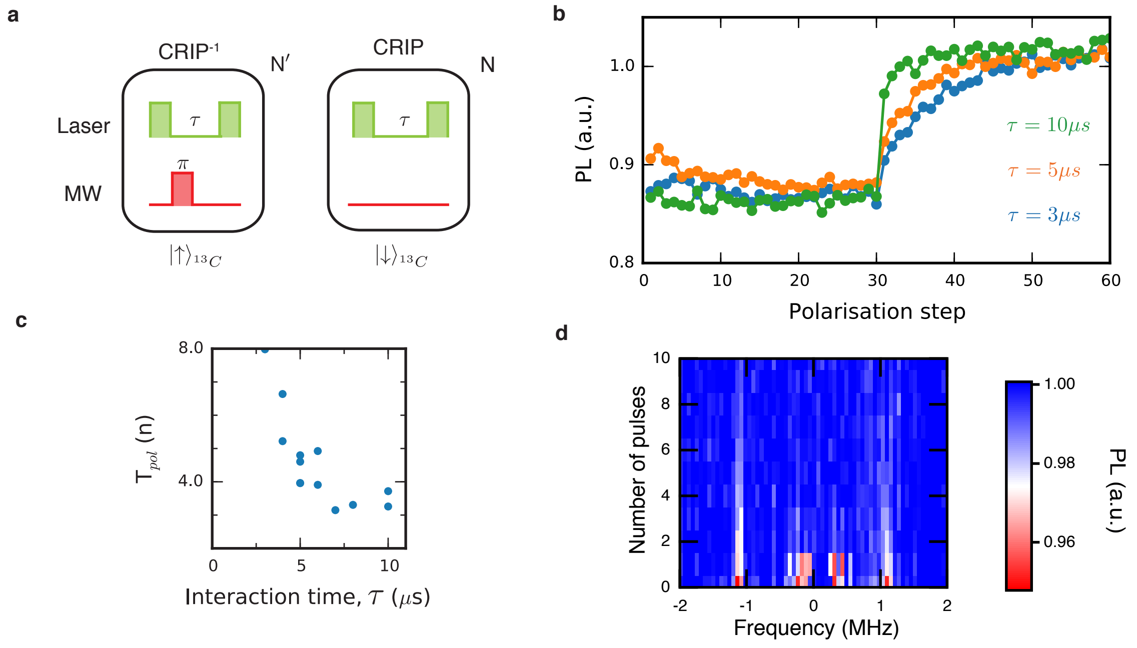

Supplementary Figure 7. **Polarisation dynamics. a** Pulse sequence used to investigate the polarisation dynamics. **b** Full sequence of 30 CRIP^{-1} pulses followed by 30 CRIP as shown in **a**, for different interaction times τ. **c** Characteristic time for polarisation as a function of interaction time. **d** T_1-relaxometry spectrum across the GSLAC, as a function of CRIP pulse number, obtained using the sequence shown in **a** with a fixed total number of CRIP and CRIP^{-1} pulses ($N' = N = 30$). The interaction time was 3 μs. Negative frequency denotes past the GSLAC.

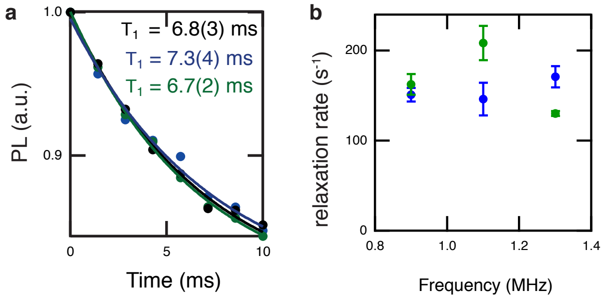

Supplementary Figure 8. **Polarisation extent measurements.** **a** Full T_1 measurements taken for the after GSLAC case in three conditions: measured on resonance with ^{13}C (blue), closer to the GSLAC i.e. ≤ 0.9 MHz (green) and further away from the GSLAC i.e. ≥ 1.3 MHz (black). **b** Decay rate the of NV around the ^{13}C resonance, before the GSLAC (green) giving $\Gamma_{\rm CR} \approx 62$ Hz and after (blue), where no change is seen within noise, giving $\Gamma_{\rm CR} \lesssim 19$ Hz.

number of polarisation pulses required as the interaction time is increased. It is important to note that due to the relatively short interaction time used in these measurements, the contrast obtained is dominated by the few nearest spins and is therefore an indication of the polarisation of nearby ^{13}C spins only, which appear to be efficiently polarised by the CRIP protocol after a few tens of μs. More remote ^{13}C spins can be probed using longer τ times, as done in main text Fig. 2b, but the significantly longer acquisition time for these measurements prevents a systematic study of the polarisation dynamics as done in Supplementary Fig. 7.

Finally in order to test the robustness of the sequence to the resonance position we performed a magnetic sweep across both resonances and the GSLAC, shown in Supplementary Fig. 7**d**. Four features are present, two related to ^{13}C (outside peaks) and two related to the GSLAC (inside peaks). The width of the polarisation region is roughly 0.1 - 0.2 MHz. The width of this polarisation region is governed by the T_2^* of the NV spin. A comparison of the number of pulses required to polarise the nearest ^{13}C spins on both sides of the GSLAC shows a distinct difference, which is explained by the difference in the angular dependence of the dipole-dipole coupling before and after the GSLAC.

Spatial extent of ^{13}C polarisation

To monitor drifts in the magnetic field (caused by thermal fluctuations), the full-length T_1 measurements used to estimate the extent of polarisation (main text Fig. 2b) were accumulated while monitoring the NV frequency via an ODMR spectrum taken every 30 minutes. In general, the NV frequency remained on resonance with the ^{13}C transition frequency (1.09 MHz) for several hours, before it drifted away by more than the NV intrinsic linewidth (≈ 200 kHz). For the data in main text Fig. 2b, the total acquisition time was 5 hours, during which the NV was on resonance within the NV linewidth, i.e. $\omega_{\rm NV}$ in the range 1.0-1.2 MHz. This ensures nearly optimal interaction between NV and ^{13}C bath, which is important not only to polarise the ^{13}C bath but also to accurately probe the remaining NV-^{13}C interaction which we used to estimate the polarisation extent. For the reference measurements off resonance, we repeated this procedure by maintaining the NV at lower frequency (< 0.9 MHz), and then at higher frequency (> 1.3 MHz), for more than 10 hours each. The final curves were then fitted with exponentials to determine the total relaxation rate $\Gamma_{\rm tot}$, which is expressed as $\Gamma_{\rm tot} = \Gamma_{\rm bg} + \Gamma_{\rm CR}$ on resonance, and simply $\Gamma_{\rm tot} = \Gamma_{\rm bg}$ off resonance. The full-length T_1 curves are shown in Supplementary Fig. 8**a** for the NV used in main text Fig. 2 near the after-GSLAC resonance, under the three conditions explained above. The extracted total decay times are shown in Supplementary Fig. 8**b** for both before and after GSLAC cases. The data before the GSLAC (green) shows a slight change in the decay rate measured on resonance, i.e. $\Gamma_{\rm CR} \approx 62$ Hz. After the GSLAC however, there is no discernible difference outside of error, $\Gamma_{\rm CR} \lesssim \Gamma_{\rm error} = 19$ Hz, given by the uncertainty of the fit parameter.

Effect of ^{13}C polarisation on NV spin coherence

As the limiting factor of the dephasing time of the NV spin (T_2^*) is noise from the ^{13}C bath (for the deep NVs considered here), it is expected that polarisation of the ^{13}C spins will result in an increase in the T_2^* of the NV. This effect was observed in previous works that employed a Hartmann-Hahn resonance to transfer polarisation Supplementary Ref. [3, 4]. To measure T_2^*, one usually performs a free induction decay (FID, or Ramsey) experiment. However, such a measurement cannot be done at the cross-relaxation resonance because it would interfere with the flip-flop dynamics

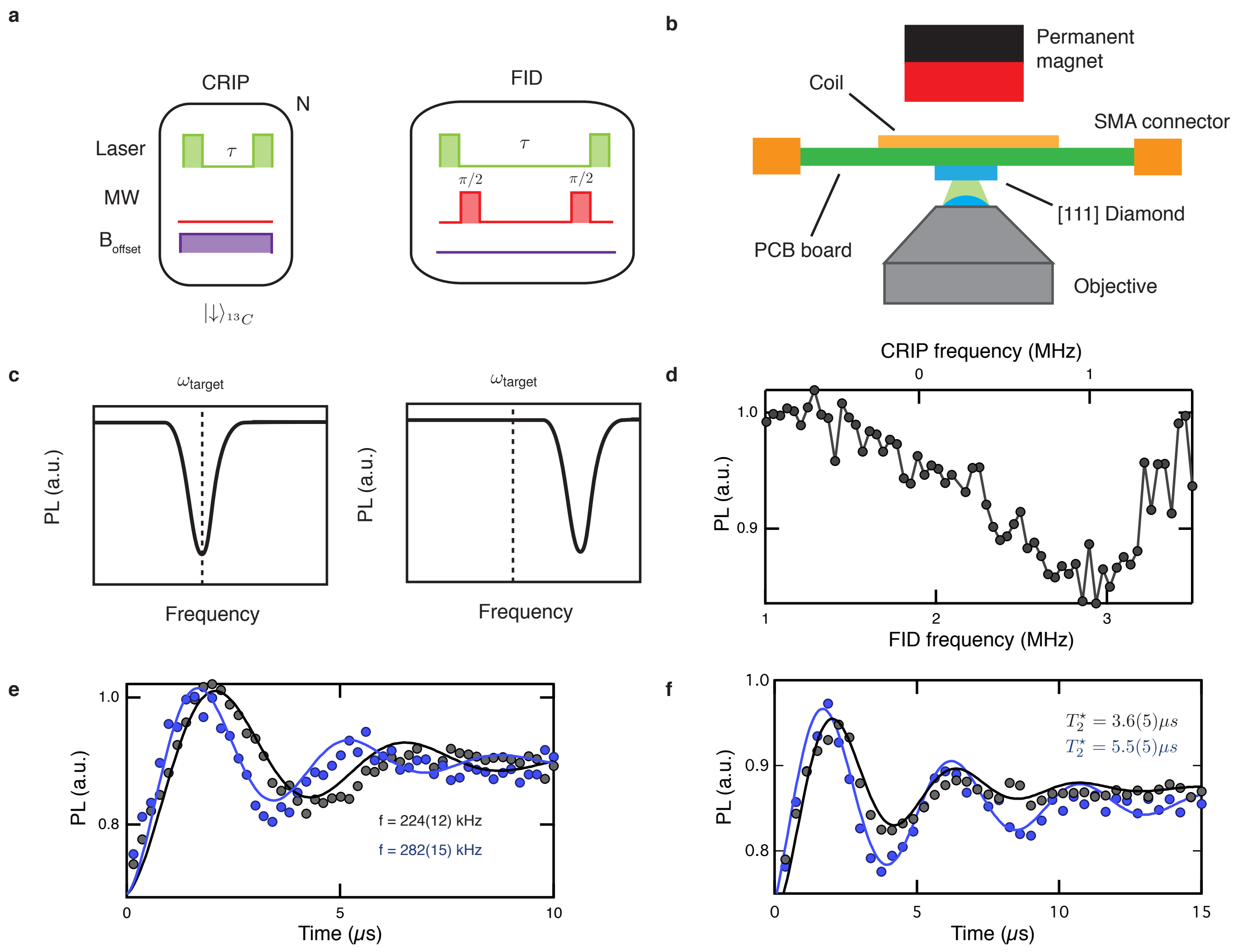

Supplementary Figure 9. **Effect of ^{13}C polarisation on the free induction decay.** **a** Pulse sequence for CRIP polarisation at the resonant frequency followed by an FID measurement at a different frequency, using a magnetic field offset pulse. **b** Schematic of the experimental set up with the coil for the field offset. **c** Example ODMR of the NV during the CRIP phase (left) and during FID measurements (right). **d** FID signal as a function of NV frequency as measured during the CRIP phase (top axis) and during the FID phase (bottom axis). The FID wait time is $\tau = 4$ μs. A detuning of 0.2 MHz was applied when driving the NV in the FID measurement. **e** FID curves obtained from a similar sweep as in **d**. The two curves show the case where the CRIP phase is on resonance (blue, polarisation) and off resonance (black, no polarisation). **f** Long FID measurement taken at a fixed frequency with the CRIP phase on resonance (blue, polarisation) and off resonance (black, no polarisation). The applied driving detuning was different in the two case in order to obtain the same oscillation frequency.

of the NV-^{13}C interaction. A solution is to polarise the bath at the resonant field then quickly shift the field to off the resonance and perform the T_2^* measurement, following the sequence shown in Supplementary Fig. 9**a**. To implement this, a coil was attached to the board holding the diamond (schematic shown in Supplementary Fig. 9**b**). A TTL pulse was used to generate a DC field from the coil to act as a quick field switching mechanism, allowing the polarisation to occur at the resonance frequency and the measurement to be performed at another frequency, as illustrated by the ODMR spectra shown in Supplementary Fig. 9**c**. The field offset thus applied was $B_{\textbf{offset}} \approx 0.7$ G, turned on while a series of polarisation pulses was implemented. The offset was then turned off and the FID measurement performed.

Using the same NV as in main text Fig. 2, we first performed a single-τ FID measurement as a function of the magnetic field, across the ^{13}C resonance (after GSLAC). The resulting spectrum is shown in Supplementary Fig. 9 **d**, where $\omega_{\rm NV}$ (measured by an ODMR spectrum at each magnet position) was scanned from -1.0 MHz to 1.5 MHz during the CRIP polarisation phase (with the field offset on), corresponding to 1 MHz to 3.5 MHz during the FID measurement (field offset off). For the polarisation phase, we used $N = 10$ CRIP pulses, and for the FID we used $\tau = 4$ μs, and a driving frequency detuned by 0.2 MHz from the NV frequency so as to induce an oscillation in the

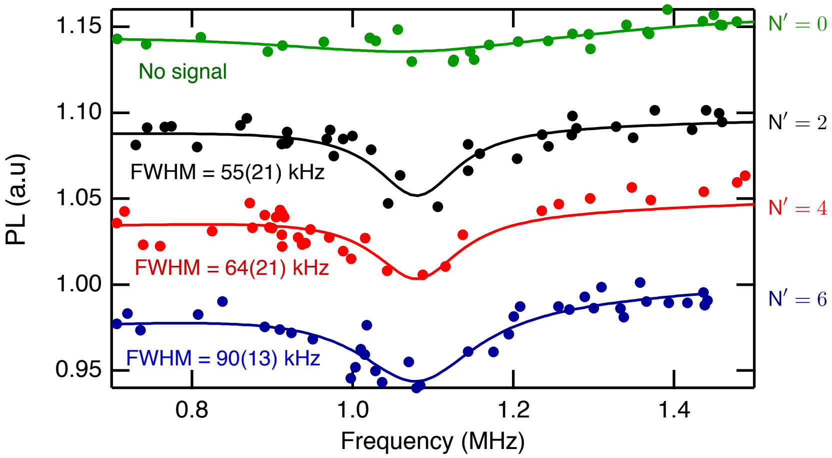

Supplementary Figure 10. **Effect of ^{13}C polarisation on the resonance linewidth.** The graph shows the ^{13}C spectrum under the sequence shown in Supplementary Fig. 7**a**, with $N = 10$ and $N' = 0, 2, 4, 6$, from top to bottom. The interaction time is $\tau = 5$ μs. A clear trend is found from fitting the signal with a Lorentzian (solid line): there is an increase in the FWHM of the feature as the bath becomes less polarised.

FID response. The CRIP+FID sequence is repeated many times at each magnet position, so that the ^{13}C bath should be efficiently polarised when on resonance. The data reveals a feature centred on the NV-^{13}C resonance, i.e. when $\omega_{\rm NV} = 1.1$ MHz during the polarisation phase. To interpret this feature, we measured full-length FID curves while scanning the magnetic field. Supplementary Fig. 9**e** shows the resulting curves with the CRIP at the resonance or off the resonance. The main difference between the two FID curves is in the frequency of the oscillation, which differ from each other by 58(27) kHz. This difference is attributed to a change in the DC magnetic field seen by the NV, induced by polarisation of the nearest ^{13}C spins. However, the envelope of the oscillation shows a similar decay time (T_2^*) in both cases, which means the ^{13}C polarisation was not sufficient to significantly reduce the magnetic noise seen by the NV. To increase the extent of the ^{13}C polarisation, we performed a longer measurement at just two fixed frequencies, on and off resonance. In addition, we adjusted the driving frequency detuning such that the FID oscillation showed the same frequency with and without polarisation, for ease of comparison. The results are shown in Supplementary Fig. 9**d**, and reveal a ∼50% increase of T_2^* from 3.6(5) μs to 5.5(5) μs upon polarisation, similar to previous results with Hartmann-Hahn based polarisation Supplementary Ref. [3, 4]. The improvement is limited by the stability of the resonance condition during CRIP, as the switching of the magnetic field to bring the NV into resonance is non ideal with our current setup (in particular, showing significant overshoot).

Another way to probe a change in T_2^* is by examining the width of the resonance feature in the T_1-relaxometry spectrum, since it is directly given by the dephasing rate $1/T_2^*$ Supplementary Ref. [2]. To test this, we implemented the sequence of Supplementary Fig. 7**a**, with $N = 10$ and a varying number of CRIP^{-1} pulses, N'. The results are shown in Supplementary Fig. 10. When $N' = 0$, there is no signal as the ^{13}C bath is polarised. As N' is increased, the width of the feature increase from 55(12) kHz for $N' = 2$ to 90(13) kHz for $N' = 6$. No difference in the width of the feature was observed for larger values of N'. The FWHM was extracted from a Lorentzian fit with a slight linear background to account for the tail of the GSLAC feature.

^{1}H polarisation

The polarisation of the ^{1}H spin bath in PMMA was performed on multiple shallow NVs. Supplementary Fig. 11**a** shows a spectrum showing the polarisation effect for a different NV (NV2) than that used in main text Fig. 3. Similarly, no ^{1}H signal was observed for the polarised sequence (blue) using an interaction time $\tau = 20\mu$s, whereas a signal is observed when the depolarisation sequence is used. We note that no measurable ^{1}H signal was observed in the absence of PMMA (removed with dichloromethane) even with the depolarisation sequence, and that the polarisation effect was observed again after reapplying PMMA. This suggests that the ^{1}H spins we detect and polarise are mainly from the PMMA, although we cannot exclude some contribution from contaminants trapped under the PMMA, e.g. water.

Like for ^{13}C, the full T_1 measurements at the ^{1}H resonance were obtained by regularly monitoring the NV frequency

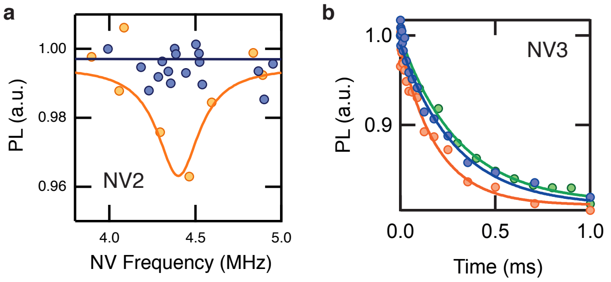

Supplementary Figure 11. **Additional ^{1}H polarisation measurements.** **a** Spectrum of ^{1}H unpolarised (orange) and polarised (blue) for NV2. **b** Full-length T_1 measurements for NV3 on resonance unpolarised (orange), polarised (blue) and a background off resonance measurement (green).

to make sure it remains on resonance within the NV linewidth ($\approx$ 500 kHz here). Because the T_1 time of shallow NVs is shorter than for bulk NVs, we could limit the total acquisition time to about 30 minutes per T_1 curve. To compare with the background T_1, the measurements were done on resonance ($\omega_{\rm NV} \approx 4.4$ MHz) and off resonance ($\omega_{\rm NV} \approx 3.0$ MHz). Supplementary Fig. 11**b** shows full-length T_1 curves from an additional NV (NV3). The data fit resulted in a ratio of relaxation rates of $\Gamma_{\rm CR}^{\rm unpol}/\Gamma_{\rm CR}^{\rm pol} = 2.4(3)$ which is consistent with the measurement from NV1 presented in the main text of 2.8(3). Error is given by the uncertainty in the decay rate fit parameter.

Supplementary References

[1] Broadway, D. A. *et al.* Anticrossing Spin Dynamics of Diamond Nitrogen-Vacancy Centers and All-Optical Low-Frequency Magnetometry. *Physical Review Applied* **6**, 064001 (2016).

[2] Hall, L. T. *et al.* Detection of nanoscale electron spin resonance spectra demonstrated using nitrogen-vacancy centre probes in diamond. *Nature Communications* **7**, 10211 (2016).

[3] London, P. *et al.* Detecting and Polarizing Nuclear Spins with Double Resonance on a Single Electron Spin. *Physical Review Letters* **111**, 067601 (2013).

[4] Liu, G.-Q. *et al.* Protection of centre spin coherence by dynamic nuclear spin polarization in diamond. *Nanoscale* **6**, 10134–9 (2014).
